# Pre-Transplant Prediction of Acute Graft-versus-Host Disease Using the Gut Microbiome

**DOI:** 10.3390/cells11244089

**Published:** 2022-12-16

**Authors:** Ramtin Zargari Marandi, Mette Jørgensen, Emma Elizabeth Ilett, Jens Christian Nørgaard, Marc Noguera-Julian, Roger Paredes, Jens D. Lundgren, Henrik Sengeløv, Cameron Ross MacPherson

**Affiliations:** 1 Centre of Excellence for Health, Immunity and Infections (CHIP), Rigshospitalet, Copenhagen University Hospital, 2100 Copenhagen, Denmark; 2 Department of Clinical Medicine, Faculty of Health and Medical Sciences, Copenhagen University, 2200 Copenhagen, Denmark; 3 IrsiCaixa Institute for AIDS Research, 08916 Badalona, Catalonia, Spain; 4 Centre for Health and Social Care Research (CESS), Faculty of Medicine, University of Vic—Central University of Catalonia (UVic—UCC), 08500 Vic, Spain; 5 Infectious Disease Networking Biomedical Research Center, Centro de Investigación Biomédica en Red de Enfermedades Infecciosas (CIBERINFEC), Carlos III Health Institute, 28029 Madrid, Spain; 6 Department of Hematology, Rigshospitalet, Copenhagen University Hospital, 2100 Copenhagen, Denmark

**Keywords:** human gut microbiome, aGvHD biomarkers, metagenome-wide association, next generation sequencing, pre-transplant screening, taxonomic assignment, deep learning, allo-HSCT

## Abstract

Gut microbiota is thought to influence host responses to allogeneic hematopoietic stem cell transplantation (aHSCT). Recent evidence points to this post-transplant for acute graft-versus-host disease (aGvHD). We asked whether any such association might be found pre-transplant and conducted a metagenome-wide association study (MWAS) to explore. Microbial abundance profiles were estimated using ensembles of Kaiju, Kraken2, and DeepMicrobes calls followed by dimensionality reduction. The area under the curve (AUC) was used to evaluate classification of the samples (aGvHD vs. none) using an elastic net to test the relevance of metagenomic data. Clinical data included the underlying disease (leukemia vs. other hematological malignancies), recipient age, and sex. Among 172 aHSCT patients of whom 42 developed aGVHD post transplantation, a total of 181 pre-transplant tool samples were analyzed. The top performing model predicting risk of aGVHD included a reduced species profile (AUC = 0.672). Beta diversity (37% in Jaccard’s Nestedness by mean fold change, *p* < 0.05) was lower in those developing aGvHD. Ten bacterial species including *Prevotella* and *Eggerthella* genera were consistently found to associate with aGvHD in indicator species analysis, as well as relief and impurity-based algorithms. The findings support the hypothesis on potential associations between gut microbiota and aGvHD based on a data-driven approach to MWAS. This highlights the need and relevance of routine stool collection for the discovery of novel biomarkers.

## 1. Introduction

Acute graft-versus-host disease (aGvHD) is a frequent serious complication in patients undergoing allogeneic hematopoietic stem cell transplantation (aHSCT) [1]. In aGvHD, immune cells from the donor attack healthy tissue of the patient, causing an increased risk of morbidity and mortality. aHSCT offers a potential cure for patients suffering from hematological diseases (most commonly leukemia), where other treatment options have been exhausted or are not suitable to tackle the patient’s aggressive disease. Finding ways to predict aGvHD prior to aHSCT in recipients is an important and still open research question [2].

With the advent of high throughput sequencing the potential role of the gut microbiome in human disease can be better understood. It is suggested that the risk of developing aGvHD could, in part, be associated with the recipients’ genetic characteristics and/or cohabitating microorganisms within the gut microbiome [3]. The gut microbiome offers an intriguing source of information having recently been suggested to improve aGvHD prediction models [4,5]. Previous studies, mainly using post-transplant samples, have found that abundancies of specific gut bacteria may provide useful information in predicting aGvHD [6]. Likewise, several gut bacteria have been associated with an increased risk of aGvHD and mortality [2]. Based on previous findings suggesting associations between the gut microbiome and aGvHD [5,7,8], we hypothesized that potential pre-aHSCT biomarkers of aGvHD could be discovered through a metagenome-wide association study (MWAS).

To test this hypothesis, we focused on species abundances with respect to prospective aGvHD occurrence. We used three taxonomic classifiers: DeepMicrobes [9], Kraken2 [10], and Kaiju [11] based on a custom database of the human gut bacteria to estimate species abundances using high throughput shotgun sequencing data from stool samples of aHSCT recipients. There was a pre-processing step to choose a configuration to aggregate metagenomic data derived from the taxonomic classifiers for downstream analyses. We used a broad set of statistical and information-theoretic methods to assess potential associations between gut species abundances and aGvHD.

## 2. Materials and Methods

### 2.1. Data Collection and Categorization

**Figure 1:**
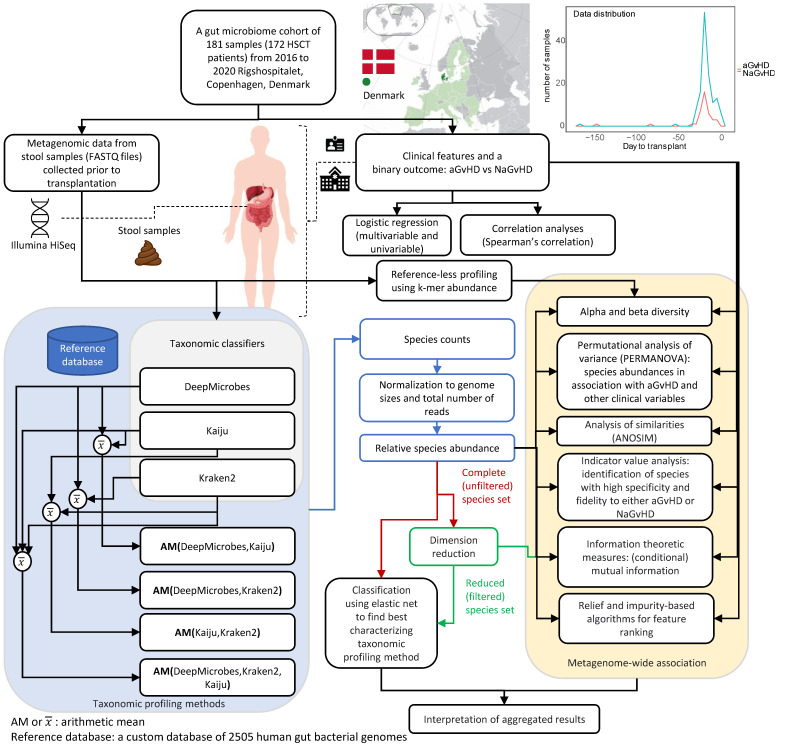
An overview of the study depicting the data distribution for stool samples with transplantation day as reference (day 0). It also depicts the focus of the study and the methodology to benchmark taxonomic profiling configurations to describe associations between gut microbes before allogeneic hematopoietic stem cell transplantation (aHSCT) and occurrence of acute graft versus host disease (aGvHD). The blue block represents the taxonomic classifiers and their combined taxonomic profiles extracting species counts. Species counts are normalized, and two sets of species are analyzed: (1) unfiltered or complete species set and (2) filtered or reduced species set. The species set with the highest classification performance is chosen for metagenome-wide association study (the block in light gold color).

Stool samples were collected by the patient or nursing staff using the OMNIgene.GUT (DNA Stabilized-frozen Inc., Ottawa, Canada) stabilization tube according to the manufacturer’s instructions. All samples were frozen a maximum of eight days after sampling and stored at −80 °C until shipment for sequencing. Paired-end sequencing (150 bp) was done using shotgun metagenomic sequencing—Illumina HiSeq (Illumina, San Diego, CA, USA). The sequencing reads underwent pre-processing and quality control steps. Using *Trimmomatic* [12] and the Illumina HiSeq program, certain adapters were eliminated. Additionally, *Trimmomatic* was used for quality trimming, that is, reads were cut if the average quality of 4 nucleotides was below 30 and nucleotides were trimmed from the start or end of the read if they had a quality score of less than 30. It also included removal of reads with <50 base pairs and removal of all reads mapping to the human genome (GRCh38) using Bowtie2 [13]. Samples with <1 million reads remaining after quality control steps were removed from further analysis. Samples were categorized into two groups depending on whether the patient developed aGvHD (grades 2 and above) or not, post transplant (i.e., an aGvHD group and a non-aGvHD (NaGvHD) group, respectively). A graphical summary of the cohort and methodology of the study is provided in Figure 1.

### 2.2. Data Analysis

The data analyses for MWAS were performed in R [14] (v4.1.2). The analyses included exploring potential associations between clinical variables including prospective aGvHD occurrence and (normalized) species abundances. Statistical significance was considered throughout this work at a level of 5% unless otherwise noted. Due to the limited sample size and our purpose of exploring a large number of candidate species in some of the analyses, the *p* values before adjustments for multiple testing are also reported.

### 2.3. Clinical Variables

We used univariable and multivariable logistic regression to find statistically significant associations of the clinical variables, i.e., conditioning regimen (myeloablative or non-myeloablative), cyclophosphamide (used or not), disease groups (acute leukemia or other hematological malignancies), donor-recipient relationship (related or unrelated), donor sex, Karnofsky score at day 0 (<90 or ≥90), radiation groups (none or irradiated), recipient age (<60 or ≥60), recipient sex, transplant source (peripheral blood or bone marrow) with aGvHD as a binary outcome variable (NaGvHD = 0, aGvHD = 1). Myeloablative is a harsher form of conditioning including high-dose cytotoxic treatment, resulting in patients becoming neutropenic in the first few weeks post-transplant, whereas non-myeloablative is less harsh, resulting in higher levels of neutrocytes post-transplant [15]. For the analysis, Karnofsky score at day 0 was imputed for two patients with the median value of it across patients with the same outcome. The dichotomization of Karnofsky score and recipient age was applied to balance their distributions by aGvHD for potential deviation from normality.

### 2.4. Taxonomic Classification

Three taxonomic classifiers, i.e., DeepMicrobes (v1.0.0), Kraken2 (v2.1.2), and Kaiju (v1.7.3) were used to assign the species from a relevant custom reference database [9] to the reads involved in each metagenomic sample. The database contains 2505 bacterial species from a bacterial repertoire of human gut microbiota. These taxonomic classifiers use different methods to assign the species to reads. The main difference is that DeepMicrobes is based on deep learning and does not require phylogenetic inference for the classification as a key advantage [9]. We extracted three species abundance profiles given the three taxonomic classifiers that were used for the downstream analyses. The taxonomic classifiers provided species counts in each sample. The species counts from the three taxonomic classifiers were aggregated by arithmetic mean (AM) leading to four extra sets: AM(DeepMicrobes, Kraken2), AM(DeepMicrobes, Kaiju), AM(Kraken2, Kaiju), and AM(DeepMicrobes, Kraken2, Kaiju). The species counts were normalized to their genome sizes and total number of reads to compute (relative) species abundances to allow their comparisons across samples (see Supplementary Material). The methodological variance unravels potential effects of taxonomic classification on distinguishing samples labeled as aGvHD from NaGvHD. As a naming protocol, the name of the most recent taxa to each species is indicated followed by “|” and the taxa identifier from the reference database. The initials refer to the taxa ranks from which the name comes from (if any): s_: species, g_: genus, o_ order, f_:family, p_:phylum, c_:class.

### 2.5. Benchmarking the Taxonomy Profiling Configurations

The samples were classified to aGvHD and NaGvHD using an elastic net in a 2 × 5-fold cross validation scheme in which the area under the curve (AUC) for receiver operating characteristic (AUROC) and precision-recall curves are reported to assess the predictability of aGvHD using each of the seven taxonomy profiling configurations. The classification was performed using SIAMCAT package (v1.14.0). We applied PERFect: PERmutation Filtering test [16], an unsupervised dimension reduction method, to find a reduced (filtered) species set in comparison with the complete (unfiltered) species set (2505 species from the database) to check whether the filtering increases signal to noise ratios quantified by AUC. This determines the taxonomy profiling configuration with the highest AUC to present the results for the downstream analyses.

### 2.6. Diversity Measures

We computed seven alpha and 10 beta diversity indices derived from species abundances as proxies to explore the patients’ gut ecosystems. Alpha diversity indices specify how diverse the species communities are within each sample, whereas beta diversity indices quantify the community diversities between samples of the aGvHD and NaGvHD groups. Alpha diversity indices were Berger-Parker dominance, Simpson’s dominance, Heip’s evenness, inverse Simpson, richness, Shannon diversity, and Strong’s dominance as implemented in abdiv package (v0.2.0). Beta diversity indices were Canberra distance, Chebyshev distance, Clark’s coefficient of divergence, correlation distance, Euclidean distance, Geodesic metric, Hellinger distance, Horn-Morisita index, Jaccard nestedness, and Manhattan distance. We also computed F_0_ [17] as a k-mer based measure of alpha diversity in addition to the mentioned alpha diversity indices. The differences in the indices were tested for statistical significance using Wilcoxon rank-sum test followed by BH adjustment [18] of the *p* values. To assess bias possibility in group difference test of significance for beta diversity indices due to different sample sizes, the test was conducted also for 1000 random subsets of the group with larger sample size against the group with smaller sample size (group-equalized testing).

### 2.7. Dissimilarity Measures

Species abundances were used to compare microbial dissimilarities in association with the occurrence of aGvHD. The distances between all pairs of samples were computed using vegdist function for “robust Aitchison” and “Bray–Curtis” measures. Analysis of similarities (ANOSIM) test using vegan package (v2.6.2) was conducted with 10,000 permutations for statistical significance of the dissimilarity measures. Permutational multivariate analysis of variance (PERMANOVA) using adonis2 function from vegan package (v2.6.2) was also performed with 10,000 permutations to assess the between-group dissimilarity of the relative abundances of samples based on the distance measures. The contributions of disease group, recipient age and sex to bacterial community composition were determined with PERMANOVA. The betadisper function was used to test the assumption of homogeneity of multivariate dispersion. To partly compensate for the sensitivity to unbalanced designs [19], the PERMANOVA and ANOSIM tests with robust Aitchison distance were also conducted for an adjusted balance setting based on Majority Weighted Minority Oversampling TEchnique (MWMOTE) algorithm [20] implemented by R package imbalance (v1.0.2.1), in which, oversampling is applied to the group with smaller sample size to generate synthetic samples resembling the distributions of the original samples.

In addition, indicator species analysis was performed using indicspecies package (v1.7.12) on species abundances to identify the species available more frequently in one group (aGvHD) compared to NaGvHD [21]. Using this analysis, group-equalized point-biserial correlation coefficients (r.g) and indicator value (IndVal.g) were used to identify the species that could be used to distinguish groups [22,23]. IndVal.g quantifies associations between aGvHD and species abundances by indicating both fidelity (sensitivity or coverage within a group) and specificity (being exclusive to a group). The specificity and fidelity are two quantities acting as probabilities ranging from 0 to 1 in which the higher the value represents higher probability of a species being exclusively found in a specific group and the species being found in all samples of the group. Furthermore, a permutation-based test (10,000 permutations) implemented by signassoc function was applied to identify the species with higher abundance in the aGvHD group compared to NaGvHD. The adjusted *p* values by BH or Sidak’s method for multiple testing are also reported.

### 2.8. Information-Theoretic Measures

Mutual information as implemented in infotheo package (v1.2.0.1) was used to quantify the association between species abundances and the occurrence of aGvHD conditioned by statistically significant confounders determined by PERMANOVA. This quantity allows measuring an estimated amount of information species abundances may contain with respect to the outcome given that the significant confounders are known.

### 2.9. Relief and Impurity-Based Algorithms for Metagenomic Feature Ranking

We used 13 feature ranking (selection) algorithms to identify most relevant features (species) with respect to our outcome (aGvHD vs. NaGvHD). The algorithms are based on extensions to Relief [24], impurity [25] or their combinations all implemented in CORElearn package (v1.56.0). The Relief-based algorithms differ on parameters such as number of neighbors (K) and strategies to calculate feature importance [26], all of which, lead to the following algorithms: equally assigned weights to K nearest neighbors (ReliefFequalK), exponentially decreasing weights with increasing ranks (ReliefFexpRank), K selection per feature (ReliefFbestK), myopic ReliefF (MyopicReliefF), merit-based ReliefF (ReliefFmerit), and weighting by inverse distance to selected instance (ReliefFdistance) in addition to the original Relief algorithm (Relief). The impurity-based algorithms are gain ratio (GainRatio), minimum description length (MDL), DKM index named after its authors (Dietterich, Kearns, and Mansour), information gain with uniform priors (UniformInf), Hellinger distance with equal weighting (EqualHellinger), and Euclidean distance (DistEuclid). The median rank of each species across algorithms was used to find the most relevant species in association with aGvHD.

## 3. Results

### 3.1. Cohort Characteristics: Clinical and Metagenomic Data

Our cohort consisted of 181 pre-transplant stool samples from 172 patients receiving aHSCT between January 2016, and August 2020 at Rigshospitalet, Copenhagen University Hospital, Denmark (Figure 1). Among the 181 samples, 42 were from patients who later developed aGvHD (grade II-IV according to the modified Glucksberg-Seattle criteria [27,28]) with a median (Q1–Q3) time from aHSCT to aGvHD of 33 (27–55) days within 100 days of post-aHSCT, where Q1 is the first quartile and Q3 is the third quartile. Of the 181 samples, 172 (95%) were collected within 30 days pre-aHSCT, with the median (Q1–Q3) sampling time being 20 (14–22) days prior to aHSCT. For an overview of patient characteristics, see Supplementary Table S1. There were nine patients (three who later developed aGvHD) who had two samples each, median 0 (range 0–61) days in between. Here, the sample closest to aHSCT was selected. For the patients with two samples on the same day, the samples were merged by the average of their normalized species abundances.

### 3.2. Clinical Variables in Association with aGvHD

Myeloablative conditioning was marginally associated with increased risk of aGvHD (*p* = 0.054) according to the multivariable logistic regression (Table 1). None of the other clinical features were found to have a statistically significant association with aGvHD. The statistical significance did not appear for the conditioning in the univariable logistic regression (Supplementary Table S2).

### 3.3. Benchmarking the Taxonomy Profiling Configurations

**Figure 2:**
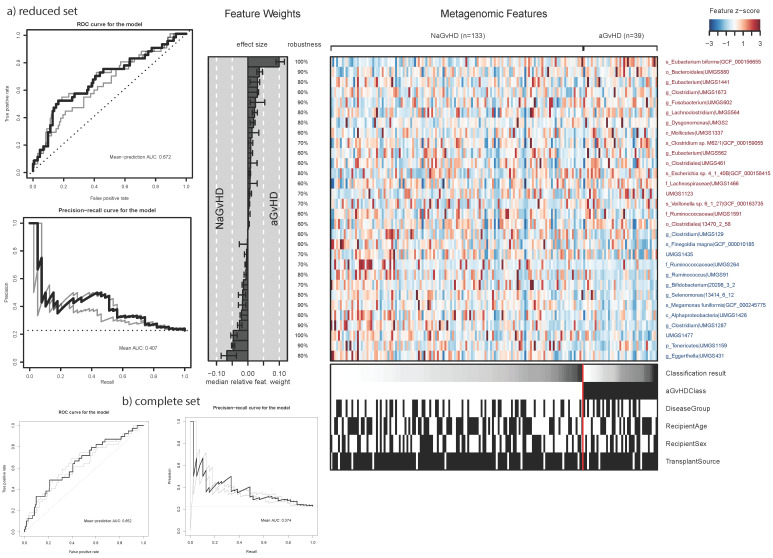
Classification performance in terms of the area under the curve (AUC) using the species abundances based on (**a**) reduced species set and (**b**) complete species set in AM(DeepMicrobes,Kaiju,Kraken2). Mean AUC (darker curves) is presented based on the cross validations in receiver operating characteristics (ROC) and precision-recall curves. On the right panel, top 20 most contributing species to the predictions according to their median weight from the elastic net model is provided as well as a heatmap of the abundances matched to the distribution of clinical features including the outcome (aGvHDClass) where black:1 and white (base):0 (levels are the same as in the PERMANOVA). The initials are the closest known taxonomy level (if any) assigned to species from the reference database, that is s_:species, g_:genus, o_:order, f_:family, and c_:class. The robustness of features refers to the percentage of times the models selected a specific feature. The result is based on N = 172 (133 NaGvHD and 39 aGvHD) samples.

The abundance of classified reads (mean% ± SD) across all three taxonomic classifiers in species level from lowest to highest were DeepMicrobes (21 ± 11), Kaiju (57 ± 7), and Kraken2 (74 ± 13). The classification performances of the best performing elastic net models based on the reduced species set and the complete species set across profiling configurations are outlined in Figure 2. The best performing models for both complete and reduced species sets were based on the combination of the three taxonomic classifiers, i.e., AM(DeepMicrobes,Kraken2,Kaiju). The highest classification performance was found for the reduced species set with AUROC = 0.672 consisting of 1443 species (57.6% of total number of species from the database). We achieved the highest performance, AUROC = 0.652 for AM(DeepMicrobes,Kraken2,Kaiju), across the seven taxonomy profiling configurations when using the complete set of species. The reduced species set had greater AUC of the precision recall curves compared with the complete set. Eubacterium biforme species was found to have the strongest association with aGvHD compared to other species (see Supplementary Figure S1). On the other hand, a species from Eggerthella genus (UMGS854) exhibited the greatest median weight towards NaGvHD in the elastic net model. The results in the following sections are based on the reduced set from AM(DeepMicrobes,Kraken2,Kaiju) as it was found to have the highest AUROC.

### 3.4. Species Diversity in Association with aGvHD

**Figure 3:**
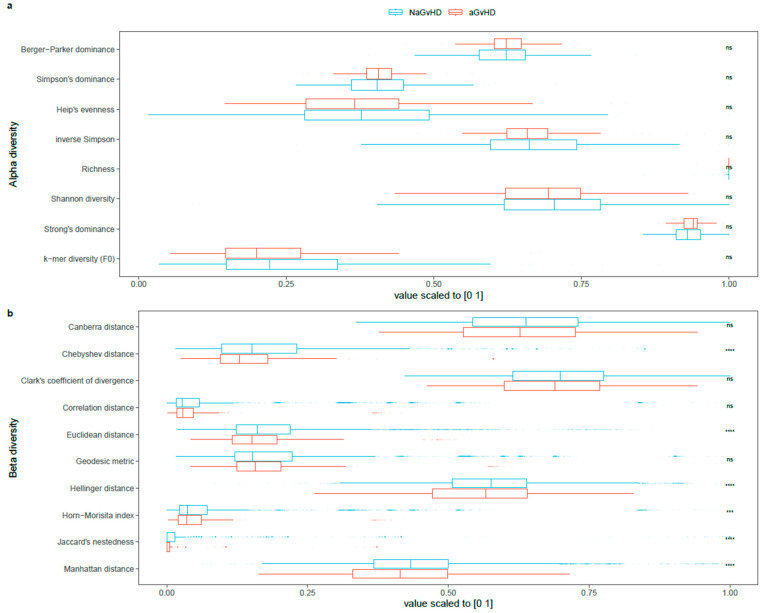
Alpha (**a**) and beta (**b**) diversity measures for AM(DeepMicrobes,Kaiju,Kraken2) with the reduced species set. Signs: *p* < 0.0001 displayed as ****, *p* < 0.001 as ***, and ns for not significant, *p* values are adjusted for multiple testing by BH adjustment. Each plot indicates the distribution of an index with a central marker for the median of the data and a box indicating the interquartile range (IQR) with tails extended to $\left(1.58\times IQR\right)/\sqrt{n}$ as 95% confidence interval where n is the number of samples. The result is based on N = 172 (133 NaGvHD and 39 aGvHD) samples.

Alpha and beta diversity indices for the aGvHD and NaGvHD groups are illustrated in Figure 3. No significant differences were found between groups in any of the alpha diversity indices, according to Wilcoxon rank-sum tests. The most distinguishing alpha diversity albeit not statistically significant was k-mer diversity (F_0_) in which greater diversity in median value for NaGvHD group exists compared to aGvHD group (approx. 14% lower in aGvHD group than the NaGvHD group by fold change of the mean values). There were statistically significant differences between groups in the beta diversity indices as outlined in 10 beta diversity indices where beta diversity was greater for NaGvHD group as compared with the aGvHD group. The greatest difference in beta diversity indices was found by Jaccard’s nestedness in which it was 37% lower in the aGvHD group by mean fold change than the NaGvHD group. By simulating group-equalized tests of significant difference between beta diversity measures of the groups, we found that the statistical significance based on adjusted *p*-values with greater diversity in the NaGvHD group existed in at least 39.4% of the random subsets for Canberra distance, and the most for 76.9% of the subsets for Chebyshev distance.

### 3.5. Dissimilarity Measures

According to PERMANOVA (Table 2), species composition was significantly explained by disease group (*p* < 0.001, 5.2%), recipient sex (*p* = 0.014, 2.1%), and aGvHD group (*p* = 0.045, 1.1%). By using Bray–Curtis distance for PERMANOVA, the statistical significance remained only for disease group (*p* < 0.001, 3.4%) and recipient sex (*p* = 0.035, 1.4%).

Oversampling of the minority group (i.e., aGvHD) resulted in an increase of N_aGvHD_ from 39 to 120 and imbalance ratio (N_aGvHD_/N_NaGvHD_ samples) from 0.29 to 0.90 (approaching to balanced design with imbalance ratio = 1). This adjustment revealed statistical significance for aGvHD group (*p* < 0.001, 2.8%), disease group (*p* < 0.001, 5.8%), recipient sex (*p* < 0.001, 2.2%), and marginally for recipient age (*p* = 0.055, 0.6%). By including interaction terms for aGvHD when the oversampling was applied in independent PERMANOVA tests, there were significant interactions between aGvHD and disease group (*p* = 0.011, 1.0%), aGvHD and recipient age (*p* = 0.016, 0.9%), as well as aGvHD and recipient sex (*p* = 0.012, 0.9%). According to the ANOSIM tests, there were significant associations of species compositions with aGvHD group only when the oversampling was applied (R = 0.031, *p* = 0.002).

**Figure 4:**
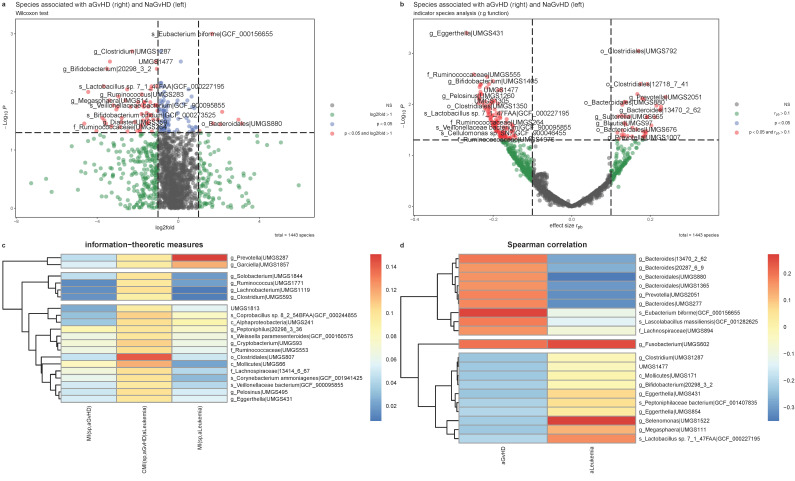
Univariable association analyses of the pre-transplant gut microbes (species abundances) with prospective aGvHD for AM(DeepMicrobes,Kaiju,Kraken2) with the reduced species set. (**a**) volcano plot using *p* values from Wilcoxon tests of significantly associated species with aGvHD where the x-axis presents effect size as log2fold change of the proportion of mean species abundance. (**b**) volcano plot depicting species potentially specific to either aGvHD or NaGvHD as found from indicator species analysis (r.g). X-axis displays the effect size in terms of point-biserial correlation. (**c**) heatmap of top 20 species found to have the highest conditional mutual information (in bits) with aGvHD conditioned by disease group (acute leukemia vs. others), (**d**) heatmap of Spearman’s correlation coefficients (rho) of the top 20 species (greatest positive and negative rho values) in association with aGvHD. *p* values on volcano plots are not adjusted for multiple testing and the labels for a subset of significant points are shown to avoid overlapping (see Supplementary Tables S3 and S4 for complete lists of significant species). The result is based on N = 172 (133 NaGvHD and 39 aGvHD) samples.

Several species were found to associate with aGvHD based on Wilcoxon’s tests (Figure 4a and Supplementary Table S3). The indicator species analysis based on r.g, also yielded the identification of 152 species in total in which 41 species were statistically significant indicators of aGvHD and 111 species being NaGvHD indicators (Figure 4b and Supplementary Table S4). Our analysis based on mutual information (Figure 4c) yielded the identification of the species *UMGS807* from *Clostridiales* order to have the highest conditional mutual information with aGvHD as conditioned by the disease group (acute leukemia vs. other hematological malignancies). The species with second highest conditional mutual information with aGvHD was *UMGS66* from *Mollicutes* class, in which it had higher mutual information with aGvHD than acute leukemia. As per Spearman correlation (Figure 4d), the species *Eubacterium biforme* had the highest positive correlation with aGvHD, and relatively low correlation with the disease group. The correlation coefficient for top 10 negatively correlated species resides in the interval of (−0.23 −0.20). The species *UMGS602* from *Fusobacterium* genus stands out in Figure 4d for being highly correlated to aGvHD and Leukemia. The species of *UMGS431* from *Eggerthella* genus was commonly found in the shortlists of top 20 highly correlated and highly associated species, respectively based on Spearman and conditional mutual information, in which it indicates that the species is more abundant in patients with no prospective aGvHD (NaGvHD).

Using the permutation-based test in species indicator analysis, we found 157 species were significantly associated with aGvHD (Supplementary Table S5). Based on the indicator species analysis using IndVal.g, 65 species were found to be significant indicator of aGvHD (Supplementary Table S6). The test was done on a filtered set of species to include species with the minimum fidelity (sensitivity) and specificity of 0.2 and 0.5, respectively, leading to 457 remaining species.

### 3.6. Relief and Impurity-Based Algorithms for Feature Ranking

**Figure 5:**
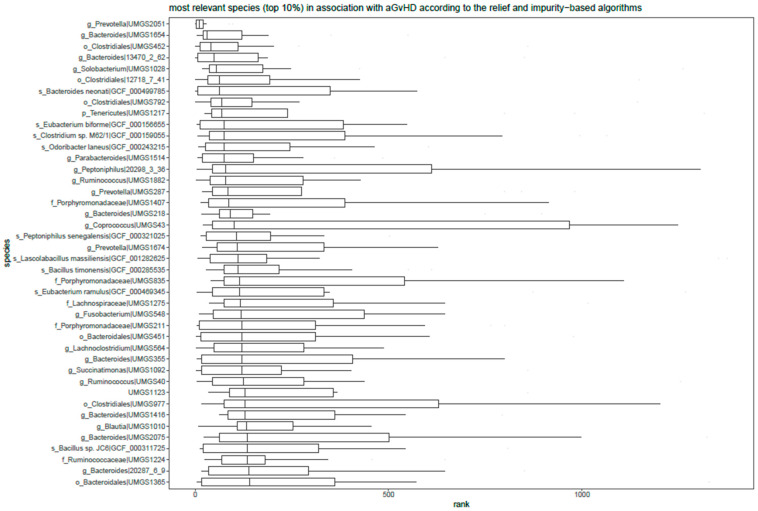
Top ranking species in association with (prospective) acute graft versus host disease (aGvHD). The species are sorted from top to bottom by their median ranks across 13 different variants of the relief and impurity algorithms. The result is based on AM(DeepMicrobes,Kaiju,Kraken2) with the reduced species set. The distribution of rankings across algorithms for each species is shown by boxplots. On the y-axis the name of the most recent taxa to each species is indicated followed by taxa identifier from the reference database. The initials refer to the taxa ranks from which the name comes from (if any): s_: species, g_: genus, o_ order, f_:family, p_:phylum. The result is based on N = 172 (133 NaGvHD and 39 aGvHD) samples.

The result of running 13 algorithms based on relief and impurity provided ranking for each species. The most relevant species in association with aGvHD are depicted Figure 5. The most relevant species from this analysis is *UMGS2051* from genus *Prevotella*.

We found 10 bacterial species that were simultaneously determined by species indicator analysis, Wilcoxon’s test, as well as relief and impurity-based algorithms to associate with aGvHD. The species are as follows: “g_Prevotella|UMGS2051”, “f_Porphyromonadaceae|UMGS211”, “g_Bacteroides|13470_2_62”, “g_Peptoniphilus|20298_3_36”, “s_Eubacterium biforme|GCF_000156655”, “s_Bacillus timonensis|GCF_000285535”, “s_Bacillus sp. JC6|GCF_000311725”, “s_Bacteroides neonati|GCF_000499785”,”s_Lascolabacillus massiliensis|GCF_001282625”, and “g_Parabacteroides|UMGS1514”. The taxa names follow the naming protocol mentioned in the method section.

## 4. Discussion

We investigated potential associations of human gut species abundances in aHSCT recipients before transplantation with subsequent occurrence of aGvHD through MWAS. We further presented candidate taxa and models that yielded the best predictive performance for aGvHD. This is the first study, to our knowledge, that utilizes metagenomics on stool samples from aHSCT patients to test the association of the abundances of gut bacteria *prior* to transplantation with prospective aGvHD occurrences. The identification of potential biomarkers of aGvHD prior to transplantation may confer significant advantage to clinical planning. We found potential prognostic biomarkers of aGvHD consistently standing out across multiple methods in MWAS.

Based on the predictive performances of the models, the reduced species set based on the ensemble method (configuration) to aggregate the taxonomic profiles seems to improve signal to noise ratio with regard to the prediction of aGvHD. This could partly be due to the different algorithms used to identify and extract species counts in which one method could outperform others depending for example on genomic characteristics of each species such as reference genome lengths. On the basis that the metagenomic samples are informative for the prediction of aGvHD, the results may suggest that the ensemble method would provide robust predictions. Notably, we used the data collected prior to transplantation whereas other studies [4,29,30] involved post transplantation data that is temporally and clinically closer to possible aGvHD incidences. The relatively small sample size and imbalanced classes were perhaps the main bottlenecks to capture stronger predictive signals, although, this does not demolish the relative strength in the power gained from gut flora measures. In a future study focused on machine learning, more complex models could be utilized to explore the possibility to improve current prediction performance and develop a deployable model.

We found conditioning being associated with aGvHD with a marginally significant effect from the multivariable logistic regression model. It may reflect that the conditioning effect on aGvHD depends on the presence of the other covariates. The association is in agreement with previous findings in which myeloablative conditioning has been shown to increase the risk of aGvHD [31,32]. The study included a limited set of variables where for example diet and ethnicity were lacking. Along with the clinical variables, metagenomic data could be obtained in the pre- and post-aHSCT periods to reveal the role of the treatments in associations with microbial compositions, and other clinical variables. For example, in post-aHSCT period, computational components derived from metagenomic data such as metabolic functions have also shown to indicate the effect of conditioning on gut microbiome for aHSCT patients [33].

Our statistical analyses revealed several species potentially associate with aGvHD. In particular, significant differences in multiple beta diversity indices gave support to the hypothesized association of the gut microbes with aGvHD. The highest difference in alpha diversity was achieved based on the k-mer measure which underlines the importance of including reference-less methods to reduce risks of biased inference due to limited list of taxa in reference databases as a common limitation for taxonomy-based methods. Another notable finding regarding species compositions was that the gut microbiome was strongly affected by the underlying disease, that is acute leukemia. This was revealed by PERMANOVA in which the disease group significantly associated with the difference in the species abundances and the effect size was seemingly higher than the association of recipient sex with species abundances. This underlined the importance of the effect of leukemia as the underlying disease to a higher extent and recipient sex to a lesser extent in the assessment of the risk of aGvHD prior to aHSCT based on metagenomics data. The lack of significant association between the bacterial compositions and aGvHD reflected in PERMANOVA and ANOSIM, as observed mainly for Bray–Curtis distance, may not necessarily rule out the presence of individual bacteria in significant association with aGvHD. It might be explained by the sensitivity of PERMANOVA and ANOSIM to unbalanced designs. The oversampling approach as an adjustment for the unbalanced design as well as the use of robust Aitchison distance with demonstrated stability to subsetting and aggregation [34] seemed effective to reveal significant associations. As such, the oversampling results suggested presence of interactions of aGvHD with recipient sex, recipient age, and disease group on independent PERMANOVA tests. The inference on the interactions is constraint to the oversampling with limitations of synthetic data to fully represent the original samples.

There are previous findings in line with the current study in which a subset of 10 bacteria were consistently determined to associate with aGvHD. As such, bacterial species from the genera *Prevotella* [5,35], Bacteroides [36], Parabacteroides [37] were found to be associated with aGvHD. The studies referred to for the bacteria are not necessarily inclusive taking into account different names available for the same species and may require a systematic review to cover the details. For example, *Holdemanella biformis* is an alternative name for the species *Eubacterium biforme* according to national library of medicine (NCBI) that was found in this study to associate with aGvHD and has been studied in relation to antibiotic shifts in aHSCT patients [38]. Some of the species such as *Lascolabacillus massiliensis* [39] found in this studies to associate with aGvHD are discovered in recent years that may explain why they are not found in the literature frequently. Bacteria from genus *Eggerthella* has been previously shown to significantly change in abundance from pre-aHSCT to post-aHSCT in a similar population [8], this may underline the significance of the species from this genus found in relation to aGvHD. In addition, higher abundance of bacteria from genus *Fusobacterium* with its unique pattern found from the correlation analysis may occur to characterize a specific immune profile of a subset of individuals with Leukemia as underlying disease to have higher risk of the occurrence of aGvHD. The notion of statistical significance should be taken cautiously in this context with regard to false discoveries especially for the species that were not found consistently to associate with aGvHD and that calls for a larger sample size and more diverse population for generalization purposes.

## 5. Conclusions

In summary, our study provides an MWAS approach to enrich abundance data derived from taxonomic classification of the human gut microbiome to improve pre-transplant risk estimation of developing aGvHD. The results reveal the potential of the methods to process complex metagenomic data to extract relevant information regarding the associations of the gut microbiome and clinical factors in the study of aGvHD from pre-aHSCT perspective. The work provides prognostic measures of aGvHD prior to aHSCT highlighting the possibility to take advantage of this new information to improve treatment and prevention strategies. In this sense, microbial information provides an enticing opportunity to enhance individualized prognostication and stratification of aGvHD risk groups.

## Figures and Tables

**Table 1:** Multivariable logistic regression with aGvHD as dependent variable (NaGvHD:0, aGvHD:1) and clinical variables as its predictors (features). Base (reference) and alternate levels for categorical features are mentioned. Number of samples were N = 172 (N_aGvHD_ = 39, N_NaGvHD_ = 133). Model fit information: Pseudo-R² (Cragg-Uhler) = 0.07, Pseudo-R² (McFadden) = 0.04, AIC = 198.37, BIC = 232.99.

Variable	Base Level	Alternate Level	β Coefficient 95% CI	β (SE)	z	*p*	VIF
(Intercept)			−3.517 (−6.829 −0.777)	1.533	−2.295	0.022	
Conditioning	non-myeloablative	myeloablative	1.759 (0.069 3.694)	0.913	1.926	0.054	5.921
Cyclophosphamide	none	used	−0.476 (−2.662 1.638)	1.07	−0.445	0.656	3.243
Disease group	acute leukemia	other hematological malignancies	−0.235 (−1.037 0.587)	0.411	−0.571	0.568	1.154
DR relationship	related	unrelated	0.318 (−0.566 1.293)	0.469	0.677	0.498	1.065
Donor sex	male	female	0.606 (−0.358 1.59)	0.494	1.228	0.219	1.702
Karnofsky D0	<90	90≥	0.166 (−0.631 0.943)	0.399	0.417	0.677	1.075
Radiation group	none	irradiated	1.204 (−0.479 3.126)	0.907	1.327	0.184	4.983
Recipient age	<60	60≥	0.186 (−0.662 1.072)	0.439	0.423	0.672	1.388
Recipient sex	male	female	−0.29 (−1.278 0.655)	0.49	−0.592	0.554	1.709
Transplant source	bone marrow	peripheral blood	0.441 (−1.237 2.294)	0.88	0.501	0.617	1.896

**Table 2:** The results of PERMANOVA on robust Aitchison distance for AM(DeepMicrobes, Kaiju, Kraken2) with the reduced species set (1443 species) based on N = 172 (133 NaGvHD and 39 aGvHD) samples.

Variable	Df	Sum Sq	R^2^	F	*p*
aGvHD group (aGvHD vs. NaGvHD)	1	5932	0.011	2.001	0.045 *
Disease group (acute leukemia vs. other hematological malignancies)	1	28,525	0.052	9.621	<0.001 ***
Recipient age (<60 vs. 60≥)	1	4183	0.008	1.411	0.147
Recipient sex (male vs. female)	1	11,783	0.021	3.974	0.014 *
Residual	167	495,129	0.899		
Total	171	550,634	1		

*** *p* < 0.001, * *p* < 0.05.

## Data Availability

The datasets generated and analyzed during this study are derived from patients treated in Denmark. The datasets contain sensitive patient data governed by the General Data Protection Regulation (GDPR) and Danish law. Due to Danish legislation (Act No. 502 of 23 May 2018) and approvals granted by the Danish Data Protection Agency (RH-2017-67, I-suite 05320), it is not possible to upload raw data to a publicly available database. However, access to these data can be made available from the corresponding author on a reasonable request, provided a data transfer agreement is entered according to current regulations. All R packages used in this study are listed in Supplementary Table S7.

## Supplementary Materials

The supporting information can be downloaded at: https://www.mdpi.com/article/10.3390/cells11244089/s1, Figure S1: abundances of the top-20 species contributing to the prediction of aGvHD as well as their associations with aGvHD and NaGvHD quantified in terms of generalized fold change, prevalence shift, and feature AUCs based on SIAMCAT package. Species names are formatted as beginning with their closest known taxonomy level assigned to them from s_:species, g_:genus, o_:order, f_:family, and c_:class or none when there is no assigned name for the species followed by the species identifier from the reference database.; Table S1: study population including 172 patients of two groups: allogeneic hematopoietic stem cell transplantation (HSCT) recipients diagnosed with acute-versus host disease (aGvHD) and HSCT recipients without aGvHD (NaGvHD); Table S2: Univariable logistic regression with aGvHD as dependent variable (NaGvHD:0, aGvHD:1) and clinical variables as predictors (features). Base (reference) and alternate levels for categorical features are mentioned. Number of samples were N = 172 (39 aGvHD and 133 NaGvHD); Table S3: list of 143 differentially abundant species according to the Wilcoxon’s test. *p* values are not adjusted for multiple testing. Species are ordered by *p* values in ascending order; Table S4: list of 152 differentially abundant species according to indicator species analyses based on group-equalized point-biserial correlation coefficients (r.g). Species are ordered by *p* values in ascending order; Table S5: list of 157 differentially abundant species according to the permutation test against the null hypothesis that the abundance of each species is not higher in one group (aGvHD) than others (NaGvHD). *p* values are adjusted for multiple testing using Sidak’s method. Mean fold change in percentage is also presented as an effect size. Species are ordered by *p* values in ascending order; Table S6: list of 65 differentially abundant species according to indicator species analyses based on group-equalized indicator value (indval.g). Species are ordered by *p* values in ascending order; Table S7: R packages used for all the analyses described in the article. The codes were compiled based on R version 4.1.2 in RStudio.
