# Supplementary material for: Pre-Transplant Prediction of Acute Graft-versus-Host Disease Using the Gut Microbiome
Source: Cells. 2022 Dec 16;11(24):4089. doi: 10.3390/cells11244089 (PMC9776596; doi:10.3390/cells11244089)
Supplement: Supplementary file 1 [file cells-11-04089-s001.zip › cells-2072693-supplementary.pdf]

Supplementary material to **Pre-transplant prediction of acute graft-versus-host disease using the gut microbiome**

Contents

Supplementary material to **Pre-transplant prediction of acute graft-versus-host disease using the gut microbiome** 1
Supplementary methods 1
Supplementary details of the taxonomic classification methods 1
DeepMicrobes: a deep learning approach for taxonomic classification 1
Kaiju and Kraken2 2
Normalization of the species abundances 2
Supplementary figures 2
Supplementary tables 3

Supplementary methods

Metagenomic study of the gut microbiome allows analysis of the genetic contents of a sample without isolating and culturing microorganisms[1]. As a common process, a fecal sample is collected from a patient followed by extracting its sequenced reads of Deoxyribonucleic acid (DNA) fragments. Further steps include taxonomic classification (after removing human DNA reads for ethical compliance) of the resulting metagenomic samples to specify the taxa-of-origin of the microorganisms in the gut microbiome using a model mapping the reads with sequences of known taxa from a reference database.

Supplementary details of the taxonomic classification methods

Fig. 1 outlines the procedure used from stool sample collection to taxonomic classification of the metagenomic samples. There are also notions on key differences between the taxonomic classification methods used in this study with further explanations as follows.

DeepMicrobes: a deep learning approach for taxonomic classification

DeepMicrobes[2] has been found to be highly precise in taxonomic classification on species level[2]. It can classify (sequence) reads with different lengths in a metagenomic sample. Furthermore, DeepMicrobes allows taxonomic classification for newly discovered species without requiring a well-curated taxonomic tree. DeepMicrobes, as a deep neural network, is structured in four computational layers. It first translates sequences of k consecutive DNA base pairs (k-mers) to vectorized numerical representations in an embedding layer. A vocabulary, as a list of possible k-mers, is also built based on canonical k-mers, where replica and reverse complements of a sequence are removed from the vocabulary. The resulting k-mers (k=12 in our study) are fed into bidirectional long short-term memory (BiLSTM). Long short-term memory (LSTM) is useful to learn long-term dependencies in a sequential data, thus, BiLSTM is a relevant choice to read sequences of k-mers in both directions in analogy to prediction of a missing word in a sentence. The output from BiLSTM is weighted using an attention mechanism that specifies which k-mers in a sequence of k-mers contain relevant information to predict species. The process is followed by an attention layer to determine

the read parts contributing the most to prediction of species. The output of the attention layer is forwarded to a multilayer perceptron to map a weighted vector generated by the attention layer to the species of origin. Each predicted/classified species thus corresponds to each read in the input sample. Each prediction is featured by a confidence score ranging 0-100%.

The confidence score is an estimated probability of the prediction's correctness using a Softmax layer implemented as the output layer of DeepMicrobes. The number of classified species (species counts) is dependent on the confidence score threshold, in which, increased threshold leads to decreased number of classified reads. Predicted species with confidence scores of >50 for DeepMicrobes were considered as classified.

DeepMicrobes has been developed using a custom database containing 2505 human gut species from complete bacterial repertoire of human gut microbiota. Many of these species are newly discovered via metagenome assembly. DeepMicrobes was downloaded from an online repository (github.com/MicrobeLab/DeepMicrobes) and implemented using Python[3] (v3.7.4) and TensorFlow[4] (v2.1.0). The processing was performed using a local IBM high performance server (New York, United States) on two graphics processing units (Nvidia Tesla v100-SXM2-16GB) with POWER8 system architecture.

We followed the instructions of the DeepMicrobes repository to use a trained deep neural network called *attention*. In order to use this model from DeepMicrobes, *tfrec_predict_kmer.sh* function was first used for the transformation of FASTQ files to TFREC files (a binary format for TensorFlow compatibility). It was followed by the prediction of the species of the origin for the reads using *predict_DeepMicrobes.sh* function.

Kaiju and Kraken2

In addition to DeepMicrobes, two different and popular methods, namely, Kaiju[5] (v1.7.3) and Kraken2[6] (v2.1.2) were also used for the taxonomic classification. The same custom reference database from DeepMicrobes[2] was used for these two methods to compare the performance of pre-HSCT aGvHD predictions derived from DeepMicrobes' classified taxa with those of Kaiju and Kraken2. The taxonomic classification using Kraken2 and Kaiju were performed in a Linux environment on Computerome, the Danish National Computer for Life Sciences and High Performance Computing servers of PERSIMUNE/CHIP. In order to use Kraken2, we followed Kraken2 manual to build our custom database and used the confidence score of 0.1 for taxonomic classification. Similarly, Kaiju was implemented following the instructions for custom databases from its software repository.

Normalization of the species abundances

The normalization was done by dividing the counts of each species by their corresponding genome size from the reference database followed by division to total counts in that sample. The resulting values then were multiplied by 100 to sum each sample's normalized counts to 100 thereby making the samples comparable. The names for taxa features are from the custom reference database and their taxonomy information can be retrieved from ftp://ftp.ebi.ac.uk/pub/databases/metagenomics/umgs_analyses/

Supplementary figures

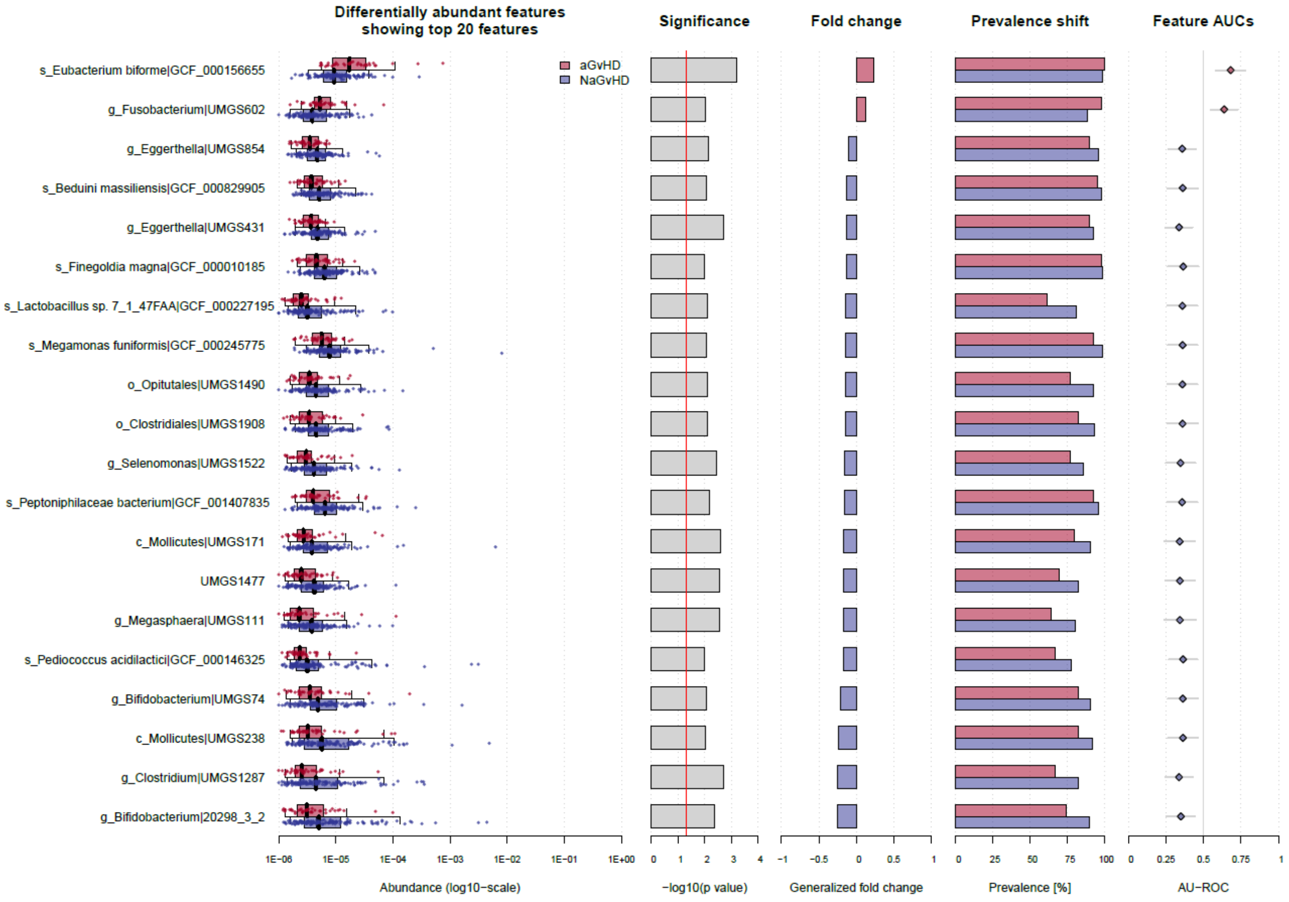

Supplementary Figure S1 abundances of the top-20 species contributing to the prediction of aGvHD as well as their associations with aGvHD and NaGvHD quantified in terms of generalized fold change, prevalence shift, and feature AUCs based on SIAMCAT package. Species names are formatted as beginning with their closest known taxonomy level assigned to them from s_:species, g_:genus, o_:order, f_:family, and c_:class or none when there is no assigned name for the species followed by the species identifier from the reference database.

Supplementary tables

Supplementary Table S1 study population including 172 patients of two groups: allogeneic hematopoietic stem cell transplantation (HSCT) recipients diagnosed with acute-versus host disease (aGvHD) and HSCT recipients without aGvHD (NaGvHD).

**Characteristic**	**N = 172**[1]
aGvHD Class	
NaGvHD	133 (77%)
aGvHD	39 (23%)
Conditioning	
myeloablative	64 (37%)
non myeloablative	108 (63%)
Cyclophosphamide	
none	155 (90%)
used	17 (9.9%)
Disease Group	
other	111 (65%)
Acute leukemia	61 (35%)
DR Relationship	
unrelated	130 (76%)
related	42 (24%)
Donor Sex	
M	109 (63%)

F	63 (37%)
Karnofsky D0	
90<	112 (65%)
90+	60 (35%)
Radiation Group	
None	48 (28%)
irradiated	124 (72%)
Recipient Age	
60<	83 (48%)
60+	89 (52%)
Recipient Sex	
M	98 (57%)
F	74 (43%)
Transplant Source	
Bone marrow	16 (9.3%)
Peripheral blood	156 (91%)

[1] n (%)

Abbreviations: D: donor, F: female, M: male, R: recipient.

Supplementary Table S2 Univariable logistic regression with aGvHD as dependent variable (NaGvHD:0, aGvHD:1) and clinical variables as predictors (features). Base (reference) and alternate levels for categorical features are mentioned. Number of samples were N=172 (39 aGvHD and 133 NaGvHD).

Feature	Base level	Alternate level	coefficient (β) 95% CI	z val.	p
Conditioning	non-myeloablative	myeloablative	0.619 (-0.104 1.343)	1.678	0.093
Cyclophosphamide	none	used	0.394 (-0.717 1.504)	0.695	0.487
Disease group	acute leukemia	other	-0.308 (-1.04 0.424)	-0.824	0.41
DR relationship	related	unrelated	0.286 (-0.584 1.155)	0.644	0.519
Donor sex	male	female	0.379 (-0.347 1.106)	1.023	0.306
Karnofsky D0	<90	90≥	0.201 (-0.538 0.939)	0.533	0.594
Radiation group	none	irradiated	-0.18 (-0.96 0.6)	-0.453	0.651
Recipient age	<60	60≥	-0.157 (-0.871 0.558)	-0.43	0.667
Recipient sex	male	female	0.164 (-0.553 0.882)	0.449	0.654
Transplant source	bone marrow	peripheral blood	-0.142 (-1.334 1.051)	-0.233	0.816

Supplementary Table S3 list of 143 differentially abundant species according to the Wilcoxon's test. P values are not adjusted for multiple testing. Species are ordered by p values in ascending order.

species[1]	p value[2]	effect size[3]
s_Eubacterium biforme|GCF_000156655	0.001	1.67
g_Clostridium|UMGS1287	0.002	-2.27
g_Eggerthella|UMGS431	0.002	-0.87
UMGS1477	0.003	-1.11
g_Megasphaera|UMGS111	0.003	0.12
c_Mollicutes|UMGS171	0.003	-3.38
g_Bifidobacterium|20298_3_2	0.004	-3.51
g_Selenomonas|UMGS1522	0.004	-1.06
s_Peptoniphilaceae bacterium|GCF_001407835	0.007	-0.87
g_Eggerthella|UMGS854	0.007	-0.84
s_Lactobacillus sp. 7_1_47FAA|GCF_000227195	0.008	-1.32
o_Opitutales|UMGS1490	0.008	-1.05
s_Megamonas funiformis|GCF_000245775	0.008	-3.68
s_Beduini massiliensis|GCF_000829905	0.008	-0.73
o_Clostridiales|UMGS1908	0.008	-0.88
g_Bifidobacterium|UMGS74	0.009	-1.35
g_Fusobacterium|UMGS602	0.009	0.50
c_Mollicutes|UMGS238	0.009	-2.53
s_Finegoldia magna|GCF_000010185	0.01	-0.70
s_Pediococcus acidilactici|GCF_000146325	0.01	-4.45

o_Clostridiales|UMGS1411	0.011	-0.89
g_Ruminococcus|UMGS283	0.011	-1.81
g_Bacteroides|13470_2_62	0.012	0.66
g_Garciella|UMGS1857	0.012	-0.70
g_Ruminococcus|UMGS91	0.012	-2.95
g_Megasphaera|UMGS14	0.014	-3.40
o_Clostridiales|UMGS207	0.014	-2.61
s_Collinsella stercoris|GCF_000156215	0.014	-0.87
s_Lascolabacillus massiliensis|GCF_001282625	0.014	0.45
g_Ruminococcus|UMGS136	0.014	-0.04
o_Clostridiales|UMGS821	0.014	-0.72
c_Clostridia|UMGS1322	0.015	-1.68
s_Weissella paramesenteroides|GCF_000160575	0.015	-1.38
s_Corynebacterium sp. SN15|GCF_900049755	0.015	-0.51
g_Ruminococcus|UMGS200	0.015	-0.79
o_Clostridiales|UMGS509	0.015	-1.99
c_Mollicutes|UMGS914	0.015	-1.69
c_Bacilli|21673_4_55	0.016	0.26
s_Bacillus timonensis|GCF_000285535	0.016	-0.89
s_Bacillus sp. B-jedd|GCF_000821085	0.016	-0.09
s_Veillonellaceae bacterium|GCF_900095855	0.017	-1.04
g_Bifidobacterium|UMGS1405	0.017	-1.17
o_Clostridiales|UMGS1470	0.017	-3.21
f_Ruminococcaceae|UMGS555	0.017	-1.10
s_Thermoactinomycetaceae bacterium|GCF_000942395	0.017	-0.59
c_Mollicutes|UMGS30	0.017	-1.79
g_Prevotella|UMGS2051	0.018	0.68
f_Ruminococcaceae|UMGS677	0.018	-2.37
o_Clostridiales|UMGS807	0.018	-1.71
g_Selenomonas|13414_6_12	0.018	-2.04
g_Megasphaera|UMGS20	0.018	-0.73
g_Treponema|UMGS419	0.018	-1.48
s_Acidaminococcus sp. HPA0509|GCF_000411395	0.018	-2.99
f_Coriobacteriaceae|UMGS165	0.018	-0.55
g_Ruminococcus|20287_6_22	0.019	0.79
f_Ruminococcaceae|UMGS1050	0.02	-3.06
o_Clostridiales|UMGS1290	0.02	-0.95
f_Succinivibrionaceae|UMGS157	0.02	-0.88
UMGS1305	0.021	-0.69
g_Anaerotruncus|UMGS177	0.021	-0.73
s_Actinomyces ihumii|GCF_001457875	0.021	-1.02
f_Veillonellaceae|UMGS1478	0.021	-0.59
g_Fusobacterium|UMGS539	0.021	-0.74
s_Bacillus sp. JC6|GCF_000311725	0.022	-0.97
g_Bacteroides|UMGS277	0.022	2.17
o_Clostridiales|UMGS46	0.023	-0.02
g_Ruminococcus|UMGS1243	0.023	-0.67
c_Mollicutes|UMGS153	0.023	-1.56
s_Parvimonas micra|GCF_000154405	0.024	-1.81
f_Erysipelotrichaceae|UMGS1249	0.024	-0.78
s_Bifidobacterium bifidum|GCF_000273525	0.025	-1.35
p_Tenericutes|UMGS1700	0.025	-1.42
g_Cryptobacterium|UMGS93	0.025	-0.66
o_Clostridiales|UMGS1034	0.026	-0.64
o_Clostridiales|UMGS83	0.026	-0.42
s_Bacillus andreraoultii|GCF_001244735	0.027	-0.69
s_Tessaracoccus massiliensis|GCF_000826065	0.028	-0.64
s_Coriobacteriaceae bacterium|GCF_000311845	0.028	-3.75
c_Clostridia|UMGS2031	0.028	0.40
f_Veillonellaceae|20298_2_31	0.028	-2.79
s_Lactobacillus brevis|GCF_000159175	0.028	-1.55
s_Bifidobacterium angulatum|GCF_001025155	0.029	0.00
c_Alphaproteobacteria|UMGS1426	0.03	-1.24
f_Ruminococcaceae|UMGS203	0.03	-0.46
f_Lachnospiraceae|UMGS894	0.03	2.97
g_Staphylococcus|12718_7_23	0.03	-1.01
o_Clostridiales|UMGS134	0.031	-0.64
c_Mollicutes|UMGS221	0.031	-1.74

c_Mollicutes|UMGS66	0.031	-3.65
o_Bacteroidales|UMGS1365	0.032	0.51
f_Ruminococcaceae|UMGS159	0.032	-1.47
s_Clostridium sp. MS1|GCF_000820705	0.032	-0.78
g_Dialister|UMGS359	0.033	-2.14
o_Clostridiales|UMGS902	0.033	-0.53
g_Peptoniphilus|20298_3_66	0.033	-0.65
g_Clostridium|UMGS1027	0.033	-2.81
g_Megasphaera|UMGS69	0.033	-0.91
o_Clostridiales|UMGS898	0.034	-1.55
g_Peptoniphilus|20298_3_36	0.034	-0.65
g_Campylobacter|UMGS142	0.035	-4.64
o_Bacteroidales|UMGS880	0.035	3.13
g_Solobacterium|UMGS1029	0.035	-0.57
f_Ruminococcaceae|UMGS131	0.035	-3.58
o_Clostridiales|UMGS1389	0.035	1.71
o_Clostridiales|UMGS214	0.035	-1.52
o_Clostridiales|UMGS285	0.036	2.07
g_Solobacterium|UMGS886	0.036	-0.48
f_Ruminococcaceae|UMGS598	0.037	-1.81
g_Veillonella|UMGS627	0.038	-0.49
g_Bacteroides|20287_6_9	0.039	0.54
g_Ruminococcus|21673_4_22	0.039	-1.85
s_Eubacterium dolichum|GCF_000154285	0.039	-3.30
o_Clostridiales|UMGS1195	0.039	-0.67
f_Ruminococcaceae|UMGS264	0.04	-2.87
o_Clostridiales|20298_3_39	0.04	-0.66
g_Parabacteroides|UMGS1514	0.04	0.86
f_Ruminococcaceae|UMGS1668	0.04	-0.92
g_Olsenella|UMGS1887	0.041	-0.65
s_Parabacteroides sp. 2_1_7|GCF_000157035	0.041	0.88
UMGS1454	0.041	0.67
g_Phascolarctobacterium|UMGS1554	0.042	-0.76
g_Ruminococcus|UMGS1601	0.042	-0.75
g_Pelosinus|UMGS1260	0.042	-0.65
s_Corynebacterium ammoniagenes|GCF_001941425	0.043	-0.90
o_Clostridiales|UMGS775	0.044	-1.88
o_Clostridiales|UMGS882	0.044	-0.82
g_Bacteroides|UMGS845	0.045	-0.06
s_Bacteroides neonati|GCF_000499785	0.045	0.69
s_Acidaminococcus sp. Marseille-P2828|GCF_900095825	0.045	-0.48
g_Actinomyces|UMGS520	0.045	-0.69
g_Clostridium|UMGS79	0.046	0.74
g_Ruminococcus|UMGS526	0.047	-2.03
o_Clostridiales|UMGS1332	0.047	1.05
g_Ruminococcus|UMGS761	0.047	-0.69
UMGS951	0.047	-4.04
g_Ruminococcus|UMGS176	0.047	-0.86
f_Ruminococcaceae|UMGS664	0.048	-2.51
s_Paenibacillus sp. HGH0039|GCF_000411255	0.048	-0.44
g_Anaerococcus|UMGS594	0.048	-1.31
f_Micrococcaceae|UMGS928	0.048	-1.89
g_Azospirillum|UMGS63	0.049	-0.20
f_Porphyromonadaceae|UMGS211	0.049	0.55
s_Numidum massiliense|GCF_001375555	0.049	-0.48

[1] Species names are formatted as beginning with their closest known taxonomy level assigned to them from s_:species, g_:genus, o_:order, f_:family, and c_:class or none when there is no assigned name for the species followed by "|" and the species identifier from the reference database.

[2] only statistically significant results (p<0.05), the statistical significance did not remain after BH adjustment for multiple testing.

[2] calculated as $\log_2(M_{aGvHD})/M_{NaGvHD})$, where M_{aGvHD} and M_{NaGvHD} are mean species abundance in aGvHD and NaGvHD groups, respectively.

Supplementary Table S4 list of 152 differentially abundant species according to indicator species analyses based on group-equalized point-biserial correlation coefficients (r.g.) Species are ordered by p values in ascending order.

**Species[1]**	**p value[2]**	**Effect size[3]**
g_Eggerthella|UMGS431	0.0004	-0.27
o_Clostridiales|UMGS792	0.0009	0.17
f_Ruminococcaceae|UMGS555	0.0026	-0.25

g_Eggerthella|UMGS854	0.0029	-0.22
g_Bifidobacterium|UMGS1405	0.0035	-0.20
o_Clostridiales|12718_7_41	0.0040	0.19
s_Eubacterium biforme|GCF_000156655	0.0040	0.18
g_Megasphaera|UMGS69	0.0040	-0.21
g_Cryptobacterium|UMGS93	0.0040	-0.23
f_Succinivibrionaceae|UMGS157	0.0042	-0.23
g_Phascolarctobacterium|UMGS1554	0.0046	-0.23
s_Corynebacterium ammoniagenes|GCF_001941425	0.0047	-0.22
UMGS1477	0.0052	-0.18
g_Streptococcus|UMGS1794	0.0053	-0.19
c_Alphaproteobacteria|UMGS1426	0.0057	-0.23
s_Collinsella stercoris|GCF_000156215	0.0063	-0.23
g_Anaerotignum|UMGS1552	0.0063	0.17
g_Selenomonas|UMGS1324	0.0064	-0.23
g_Pelosinus|UMGS1260	0.0068	-0.24
g_Prevotella|UMGS2051	0.0072	0.24
g_Blautia|UMGS1786	0.0077	0.18
s_Beduini massiliensis|GCF_000829905	0.0078	-0.23
g_Cryptobacterium|UMGS1745	0.0079	-0.24
g_Blautia|UMGS1010	0.0080	0.18
c_Mollicutes|UMGS221	0.0084	-0.20
UMGS1305	0.0086	-0.20
g_Megasphaera|UMGS20	0.0089	-0.22
s_Eubacterium ramulus|GCF_000469345	0.0090	0.14
g_Anaerococcus|UMGS594	0.0090	-0.20
g_Ruminococcus|UMGS1601	0.0091	-0.23
o_Bacteroidales|UMGS880	0.0091	0.13
s_Thalassobacillus sp. TM-1|GCF_001368835	0.0095	-0.24
s_Actinomyces ihumii|GCF_001457875	0.0095	-0.20
f_Ruminococcaceae|UMGS1668	0.0097	-0.19
g_Bacteroides|UMGS277	0.0102	0.13
s_Finegoldia magna|GCF_000010185	0.0104	-0.22
f_Succinivibrionaceae|UMGS528	0.0104	-0.21
s_Bacteroides neonati|GCF_000499785	0.0106	0.22
o_Clostridiales|UMGS1350	0.0108	-0.22
g_Bacteroides|UMGS1654	0.0111	0.23
f_Ruminococcaceae|UMGS230	0.0118	-0.22
s_Clostridiales bacterium|GCF_001282665	0.0120	-0.21
g_Clostridium|UMGS1808	0.0123	-0.21
s_Bifidobacterium breve|GCF_001025175	0.0126	-0.20
s_Bacillus sp. JC6|GCF_000311725	0.0126	-0.22
g_Parabacteroides|UMGS1514	0.0127	0.23
g_Bacteroides|13470_2_62	0.0129	0.22
s_Bacillus timonensis|GCF_000285535	0.0142	-0.21
s_Olsenella sp. KHD7|GCF_900078545	0.0143	-0.21
s_Lactobacillus sp. 7_1_47FAA|GCF_000227195	0.0149	-0.19
g_Clostridium|UMGS1287	0.0150	-0.18
f_Lachnospiraceae|13470_2_93	0.0157	-0.21
UMGS1281	0.0163	-0.20
g_Exiguobacterium|14672_4_4	0.0168	-0.21
f_Ruminococcaceae|UMGS1071	0.0168	0.13
g_Olsenella|UMGS1887	0.0169	-0.20
g_Lactobacillus|12718_7_59	0.0171	-0.19
g_Sutterella|UMGS665	0.0174	0.15
c_Alphaproteobacteria|UMGS241	0.0174	0.12
s_Lachnospiraceae bacterium 6_1_63FAA|GCF_000209425	0.0175	0.14
o_Clostridiales|14207_7_59	0.0176	-0.18
f_Lachnospiraceae|8080_1_69	0.0178	0.19
o_Clostridiales|UMGS452	0.0178	0.19
g_Parabacteroides|UMGS1201	0.0179	0.21
s_Clostridium sp. MS1|GCF_000820705	0.0180	-0.21
f_Lachnospiraceae|13414_6_33	0.0187	-0.18
g_Pelosinus|UMGS495	0.0193	-0.21
g_Lactobacillus|12718_7_14	0.0196	-0.17
s_Cetobacterium somerae|GCF_000479045	0.0199	-0.19
o_Clostridiales|20298_3_39	0.0203	-0.21
o_Clostridiales|UMGS1290	0.0209	-0.19

o_Clostridiales|UMGS1326	0.0219	-0.20
o_Clostridiales|UMGS821	0.0222	-0.19
f_Ruminococcaceae|UMGS264	0.0224	-0.19
g_Clostridium|UMGS453	0.0230	-0.20
s_Desulfitobacterium hafniense|GCF_000238035	0.0230	-0.19
g_Selenomonas|UMGS1522	0.0231	-0.17
f_Coriobacteriaceae|UMGS165	0.0232	-0.19
g_Veillonella|UMGS627	0.0232	-0.20
g_Blautia|UMGS97	0.0233	0.13
f_Erysipelotrichaceae|UMGS415	0.0235	-0.19
g_Clostridium|UMGS1027	0.0243	-0.15
p_Tenericutes|UMGS592	0.0245	-0.20
g_Dysgonomonas|UMGS2	0.0247	0.14
p_Tenericutes|UMGS483	0.0258	-0.19
f_Ruminococcaceae|UMGS1491	0.0259	-0.20
f_Porphyromonadaceae|UMGS1407	0.0261	0.20
o_Clostridiales|UMGS361	0.0261	-0.15
s_Kurthia sp. JC30|GCF_000285555	0.0266	-0.20
g_Peptoniphilus|20298_3_36	0.0271	-0.19
f_Erysipelotrichaceae|UMGS1249	0.0274	-0.20
g_Desulfovibrio|UMGS344	0.0275	-0.15
f_Veillonellaceae|UMGS1478	0.0279	-0.20
s_Veillonellaceae bacterium|GCF_900095855	0.0282	-0.17
o_Opitutales|UMGS1490	0.0285	-0.17
g_Peptoniphilus|20298_3_66	0.0287	-0.19
s_Odoribacter laneus|GCF_000243215	0.0289	0.15
g_Clostridium|UMGS101	0.0303	-0.19
f_Ruminococcaceae|UMGS1775	0.0305	-0.16
o_Bacteroidales|UMGS676	0.0311	0.16
g_Eubacterium|UMGS192	0.0317	-0.19
s_Lactobacillus brevis|GCF_001433855	0.0322	-0.15
o_Bacteroidales|UMGS764	0.0330	0.13
f_Lachnospiraceae|UMGS1615	0.0331	-0.17
f_Ruminococcaceae|UMGS1858	0.0335	-0.19
f_Porphyromonadaceae|UMGS211	0.0336	0.19
f_Lachnospiraceae|UMGS1691	0.0336	-0.19
g_Ruminococcus|UMGS739	0.0338	-0.19
f_Ruminococcaceae|UMGS664	0.0340	-0.15
g_Eubacterium|UMGS1529	0.0342	0.18
s_Lactobacillus brevis|GCF_000159175	0.0346	-0.15
o_Bacteroidales|UMGS451	0.0350	0.19
f_Rhodospirillaceae|UMGS1296	0.0351	-0.19
g_Fusobacterium|UMGS548	0.0351	-0.18
g_Bacteroides|UMGS212	0.0358	0.12
g_Lachnobacterium|UMGS1119	0.0362	-0.18
s_Cellulomonas sp. SN7|GCF_900046455	0.0364	-0.17
g_Ruminococcus|UMGS1659	0.0364	-0.17
s_Lascolabacillus massiliensis|GCF_001282625	0.0379	0.19
f_Lachnospiraceae|UMGS109	0.0382	-0.18
s_Kallipyga gabonensis|GCF_001286805	0.0390	-0.18
s_Paenibacillus sp. Marseille-P2472|GCF_900086655	0.0396	-0.18
g_Blautia|UMGS335	0.0397	0.13
o_Clostridiales|UMGS416	0.0397	-0.17
g_Bacteroides|UMGS1647	0.0398	0.13
g_Solobacterium|UMGS1469	0.0407	-0.15
g_Ruminococcus|UMGS91	0.0409	-0.15
g_Bacteroides|UMGS1416	0.0416	0.18
s_Fusobacterium mortiferum|GCF_000158195	0.0418	-0.18
s_Sutterella wadsworthensis|GCF_000186505	0.0418	-0.17
g_Fusobacterium|UMGS539	0.0419	-0.18
g_Solobacterium|UMGS1737	0.0421	0.14
o_Clostridiales|UMGS913	0.0423	-0.17
s_Paenibacillus ihumii|GCF_001403875	0.0426	-0.18
g_Paenibacillus|UMGS122	0.0426	-0.18
g_Ruminococcus|UMGS1243	0.0426	-0.18
g_Clostridium|12718_7_39	0.0429	0.15
g_Blautia|20298_3_81	0.0434	0.13
f_Lachnospiraceae|UMGS1777	0.0434	-0.18

g_Ruminococcus|UMGS1882	0.0434	0.18
g_Olsenella|UMGS1629	0.0437	-0.17
g_Phascolarctobacterium|UMGS1456	0.0438	-0.18
g_Bacillus|17138_5_75	0.0441	-0.18
g_Prevotella|UMGS1007	0.0444	0.18
g_Solobacterium|UMGS1140	0.0444	-0.15
o_Clostridiales|UMGS1908	0.0451	-0.17
c_Mollicutes|UMGS914	0.0451	-0.14
g_Clostridium|UMGS341	0.0459	-0.16
s_Bifidobacterium gallicum|GCF_000741205	0.0471	-0.16
g_Cloacibacillus|UMGS813	0.0478	-0.16
g_Odoribacter|UMGS1049	0.0483	0.12
f_Ruminococcaceae|UMGS1976	0.0487	-0.17

[1] Species names are formatted as beginning with their closest known taxonomy level assigned to them from s_:species, g_:genus, o_:order, f_:family, and c_:class or none when there is no assigned name for the species followed by "|" and the species identifier from the reference database.
[2] only statistically significant results ($p<0.05$), the statistical significance did not remain after BH adjustment for multiple testing.
[3] r.g., positive values can be interpreted as positive correlation with aGvHD and vice versa.

Supplementary Table S5 list of 157 differentially abundant species according to the permutation test against the null hypothesis that the abundance of each species is not higher in one group (aGvHD) than others (NaGvHD). P values are adjusted for multiple testing using Sidak's method. Mean fold change in percentage is also presented as an effect size. Species are ordered by p values in ascending order.

Species[1]	p sidak[2]	Mean fold change[3]	Higher abundant group
g_Eggerthella|UMGS431	0.001	-45.2	NaGvHD
o_Clostridiales|UMGS792	0.002	1160.2	aGvHD
UMGS1477	0.002	-53.7	NaGvHD
g_Eggerthella|UMGS854	0.002	-44.3	NaGvHD
o_Clostridiales|UMGS207	0.003	-83.6	NaGvHD
s_Megamonas funiformis|GCF_000245775	0.003	-92.2	NaGvHD
c_Mollicutes|UMGS221	0.003	-70.1	NaGvHD
f_Ruminococcaceae|UMGS555	0.004	-53.3	NaGvHD
s_Corynebacterium ammoniagenes|GCF_001941425	0.004	-46.4	NaGvHD
f_Ruminococcaceae|UMGS1050	0.004	-88.0	NaGvHD
g_Streptococcus|UMGS1794	0.005	-66.2	NaGvHD
g_Prevotella|UMGS2051	0.005	60.7	aGvHD
g_Megasphaera|UMGS69	0.005	-46.8	NaGvHD
g_Cryptobacterium|UMGS93	0.005	-36.8	NaGvHD
g_Lactobacillus|12718_7_14	0.006	-73.6	NaGvHD
s_Pediococcus acidilactici|GCF_000146325	0.006	-95.4	NaGvHD
g_Bifidobacterium|UMGS1405	0.006	-55.6	NaGvHD
o_Clostridiales|12718_7_41	0.006	446.0	aGvHD
f_Ruminococcaceae|UMGS677	0.006	-80.6	NaGvHD
s_Bacteroides neonati|GCF_000499785	0.007	61.4	aGvHD
g_Clostridium|UMGS1027	0.007	-85.7	NaGvHD
f_Succinivibrionaceae|UMGS157	0.007	-45.5	NaGvHD
g_Bacteroides|UMGS1654	0.007	75.9	aGvHD
g_Desulfovibrio|UMGS344	0.007	-95.8	NaGvHD
g_Anaerococcus|UMGS594	0.007	-59.6	NaGvHD
g_Parabacteroides|UMGS1514	0.008	80.9	aGvHD
g_Phascolarctobacterium|UMGS1554	0.008	-41.1	NaGvHD
c_Alphaproteobacteria|UMGS1426	0.008	-57.6	NaGvHD
s_Actinomyces ihumii|GCF_001457875	0.008	-50.8	NaGvHD
g_Pelosinus|UMGS1260	0.008	-36.1	NaGvHD
s_Beduini massiliensis|GCF_000829905	0.009	-39.8	NaGvHD
g_Parabacteroides|UMGS1201	0.010	63.2	aGvHD
g_Clostridium|UMGS1287	0.010	-79.3	NaGvHD
f_Ruminococcaceae|UMGS664	0.010	-82.4	NaGvHD
s_Collinsella stercoris|GCF_000156215	0.010	-45.1	NaGvHD
s_Eubacterium ramulus|GCF_000469345	0.010	501.2	aGvHD
g_Selenomonas|UMGS1324	0.011	-45.4	NaGvHD
g_Megasphaera|UMGS20	0.011	-39.5	NaGvHD
o_Clostridiales|UMGS452	0.011	125.3	aGvHD
UMGS1305	0.011	-38.2	NaGvHD
f_Ruminococcaceae|UMGS1668	0.012	-47.1	NaGvHD
s_Lactobacillus sp. 7_1_47FAA|GCF_000227195	0.012	-59.9	NaGvHD

g_Cryptobacterium|UMGS1745	0.013	-34.8	NaGvHD
g_Solobacterium|UMGS524	0.013	-94.4	NaGvHD
s_Lactobacillus brevis|GCF_000159175	0.013	-65.8	NaGvHD
s_Finegoldia magna|GCF_000010185	0.013	-38.3	NaGvHD
g_Anaerotignum|UMGS1552	0.013	684.9	aGvHD
g_Blautia|UMGS1786	0.013	511.5	aGvHD
o_Clostridiales|UMGS361	0.013	-66.0	NaGvHD
g_Bacteroides|13470_2_62	0.014	57.6	aGvHD
f_Lachnospiraceae|8080_1_69	0.014	222.0	aGvHD
f_Ruminococcaceae|UMGS131	0.014	-91.6	NaGvHD
s_Clostridiales bacterium|GCF_001282665	0.014	-43.3	NaGvHD
g_Blautia|UMGS1010	0.014	429.1	aGvHD
g_Ruminococcus|UMGS1601	0.015	-40.6	NaGvHD
f_Succinivibrionaceae|UMGS528	0.015	-53.4	NaGvHD
s_Thalassobacillus sp. TM-1|GCF_001368835	0.015	-34.2	NaGvHD
c_Mollicutes|UMGS914	0.015	-69.0	NaGvHD
s_Helicobacter pullorum|GCF_000155495	0.016	-70.0	NaGvHD
g_Megasphaera|UMGS14	0.016	-90.5	NaGvHD
s_Eubacterium biforme|GCF_000156655	0.017	217.6	aGvHD
g_Clostridium|UMGS1808	0.017	-48.0	NaGvHD
UMGS1281	0.018	-44.3	NaGvHD
s_Bacillus timonensis|GCF_000285535	0.018	-46.0	NaGvHD
s_Bifidobacterium breve|GCF_001025175	0.018	-72.5	NaGvHD
g_Eubacterium|UMGS1529	0.018	62.1	aGvHD
s_Lactobacillus brevis|GCF_001433855	0.018	-75.1	NaGvHD
s_Bacillus sp. JC6|GCF_000311725	0.018	-48.8	NaGvHD
g_Olsenella|UMGS1887	0.019	-36.3	NaGvHD
o_Clostridiales|UMGS1350	0.019	-43.3	NaGvHD
s_Lactobacillus helveticus|GCF_000160855	0.019	-84.7	NaGvHD
s_Olsenella sp. KHD7|GCF_900078545	0.019	-40.5	NaGvHD
f_Ruminococcaceae|UMGS230	0.019	-54.2	NaGvHD
g_Lactobacillus|12718_7_59	0.019	-62.2	NaGvHD
o_Clostridiales|14207_7_59	0.019	-75.0	NaGvHD
g_Staphylococcus|20298_3_3	0.020	-97.4	NaGvHD
f_Porphyromonadaceae|UMGS1407	0.020	58.6	aGvHD
s_Clostridium sp. MS1|GCF_000820705	0.021	-41.6	NaGvHD
g_Selenomonas|UMGS1522	0.021	-51.9	NaGvHD
g_Exiguobacterium|14672_4_4	0.022	-41.7	NaGvHD
o_Clostridiales|UMGS214	0.022	-65.1	NaGvHD
f_Lachnospiraceae|13414_6_33	0.023	-67.2	NaGvHD
g_Solobacterium|UMGS1469	0.023	-77.3	NaGvHD
g_Bacteroides|UMGS277	0.023	348.6	aGvHD
g_Pelosinus|UMGS495	0.024	-33.0	NaGvHD
o_Clostridiales|UMGS1290	0.024	-48.1	NaGvHD
s_Cetobacterium somerae|GCF_000479045	0.024	-40.1	NaGvHD
f_Coriobacteriaceae|UMGS165	0.024	-31.9	NaGvHD
o_Opitutales|UMGS1490	0.025	-51.8	NaGvHD
f_Ruminococcaceae|UMGS1775	0.025	-50.2	NaGvHD
o_Bacteroidales|UMGS676	0.025	102.9	aGvHD
f_Lachnospiraceae|UMGS794	0.025	-95.9	NaGvHD
s_Veillonellaceae bacterium|GCF_900095855	0.025	-51.2	NaGvHD
f_Ruminococcaceae|UMGS598	0.026	-71.4	NaGvHD
g_Succiniclasticum|UMGS663	0.026	-93.1	NaGvHD
o_Bacteroidales|UMGS880	0.026	774.3	aGvHD
o_Clostridiales|20298_3_39	0.026	-36.7	NaGvHD
s_Lactobacillus ultunensis|GCF_000159415	0.026	-66.6	NaGvHD
s_Acetomicrobium hydrogeniformans|GCF_000160455	0.026	-40.8	NaGvHD
g_Bacteroides|UMGS1416	0.027	86.5	aGvHD
p_Tenericutes|UMGS483	0.027	-47.3	NaGvHD
g_Veillonella|UMGS627	0.027	-28.6	NaGvHD
g_Campylobacter|UMGS142	0.027	-96.0	NaGvHD
s_Acidaminococcus sp. HPA0509|GCF_000411395	0.027	-87.4	NaGvHD
o_Clostridiales|UMGS821	0.028	-39.1	NaGvHD
f_Ruminococcaceae|UMGS264	0.028	-86.3	NaGvHD
f_Erysipelotrichaceae|20298_3_70	0.029	-67.5	NaGvHD
g_Ruminococcus|UMGS91	0.029	-87.1	NaGvHD
f_Lachnospiraceae|13470_2_93	0.029	-38.2	NaGvHD
f_Ruminococcaceae|UMGS198	0.030	-92.2	NaGvHD

f_Ruminococcaceae|UMGS1033	0.030	-95.4	NaGvHD
s_Odoribacter laneus|GCF_000243215	0.030	111.6	aGvHD
s_Lascolabacillus massiliensis|GCF_001282625	0.031	36.2	aGvHD
s_Campylobacter sp. 10_1_50|GCF_000238755	0.032	-99.3	NaGvHD
f_Erysipelotrichaceae|UMGS1249	0.032	-41.8	NaGvHD
s_Desulfitobacterium hafniense|GCF_000238035	0.033	-32.5	NaGvHD
f_Erysipelotrichaceae|UMGS415	0.034	-48.9	NaGvHD
g_Prevotella|UMGS1430	0.034	139.3	aGvHD
o_Clostridiales|UMGS1326	0.034	-39.7	NaGvHD
p_Tenericutes|UMGS592	0.034	-48.2	NaGvHD
f_Ruminococcaceae|UMGS1491	0.034	-38.1	NaGvHD
o_Clostridiales|UMGS1011	0.034	-86.4	NaGvHD
f_Veillonellaceae|UMGS1478	0.034	-33.7	NaGvHD
g_Clostridium|UMGS453	0.035	-50.1	NaGvHD
g_Cloacibacillus|UMGS813	0.036	-46.7	NaGvHD
s_Coriobacteriaceae bacterium|GCF_000311845	0.036	-92.6	NaGvHD
s_Lachnospiraceae bacterium 6_1_63FAA|GCF_000209425	0.036	990.2	aGvHD
f_Ruminococcaceae|UMGS1071	0.036	817.6	aGvHD
g_Ruminococcus|UMGS1882	0.036	66.1	aGvHD
f_Porphyromonadaceae|UMGS211	0.037	45.9	aGvHD
g_Blautia|UMGS1359	0.037	99.3	aGvHD
g_Bacteroides|UMGS901	0.037	60.1	aGvHD
o_Bacteroidales|UMGS451	0.037	34.8	aGvHD
c_Alphaproteobacteria|UMGS241	0.038	3243.6	aGvHD
f_Lachnospiraceae|UMGS1615	0.038	-41.4	NaGvHD
g_Lactobacillus|12718_7_17	0.039	-84.2	NaGvHD
s_Kurthia sp. JC30|GCF_000285555	0.039	-46.9	NaGvHD
g_Blautia|UMGS276	0.040	143.7	aGvHD
g_Treponema|UMGS419	0.040	-64.1	NaGvHD
c_Clostridia|UMGS1322	0.040	-68.7	NaGvHD
f_Ruminococcaceae|UMGS1858	0.040	-31.8	NaGvHD
g_Peptoniphilus|20298_3_36	0.040	-36.2	NaGvHD
g_Peptoniphilus|20298_3_66	0.041	-36.4	NaGvHD
f_Rhodospirillaceae|UMGS1296	0.041	-39.5	NaGvHD
g_Bifidobacterium|20298_3_2	0.041	-91.2	NaGvHD
g_Clostridium|UMGS101	0.042	-35.9	NaGvHD
g_Prevotella|UMGS1007	0.042	51.6	aGvHD
g_Ruminococcus|UMGS1243	0.043	-37.0	NaGvHD
g_Sutterella|UMGS665	0.043	1380.8	aGvHD
f_Lachnospiraceae|UMGS1691	0.044	-38.4	NaGvHD
g_Ruminococcus|UMGS526	0.044	-75.4	NaGvHD
o_Clostridiales|UMGS1908	0.045	-45.6	NaGvHD
o_Clostridiales|UMGS416	0.045	-68.3	NaGvHD
g_Blautia|UMGS97	0.047	836.2	aGvHD
g_Clostridium|UMGS341	0.047	-90.8	NaGvHD
o_Clostridiales|UMGS913	0.047	-67.6	NaGvHD
g_Coprococcus|UMGS43	0.048	-88.9	NaGvHD

[1] Species names are formatted as beginning with their closest known taxonomy level assigned to them from s_:species, g_:genus, o_:order, f_:family, and c_:class or none when there is no assigned name for the species followed by "|" and the species identifier from the reference database.

[2] only statistically significant results (p<0.05), the statistical significance did not remain after BH adjustment for multiple testing.

[2] calculated as $((M_{aGvHD}-M_{NaGvHD})/M_{NaGvHD})*100$, where M_{aGvHD} and M_{NaGvHD} are mean species abundance in aGvHD and NaGvHD groups, respectively.

Supplementary Table S6 list of 65 differentially abundant species according to indicator species analyses based on group-equalized indicator value (indval.g). Species are ordered by p values in ascending order.

Species[1]	Specificity	Fidelity (Sensitivity)	$\sqrt{indval.g}$	p value[2]
o_Clostridiales|UMGS792	0.93	1.00	0.96	0.0009
g_Bacteroides|UMGS1654	0.64	1.00	0.80	0.0024
o_Clostridiales|12718_7_41	0.85	1.00	0.92	0.0029
s_Bacteroides neonati|GCF_000499785	0.62	1.00	0.79	0.0030
g_Parabacteroides|UMGS1514	0.64	1.00	0.80	0.0033
g_Prevotella|UMGS2051	0.62	0.95	0.76	0.0041
g_Parabacteroides|UMGS1201	0.62	1.00	0.79	0.0055
o_Clostridiales|UMGS452	0.69	1.00	0.83	0.0058
g_Blautia|UMGS1010	0.84	1.00	0.92	0.0064
g_Blautia|UMGS1786	0.86	1.00	0.93	0.0068
g_Anaerotignum|UMGS1552	0.89	1.00	0.94	0.0069

f_Lachnospiraceae|8080_1_69	0.76	1.00	0.87	0.0070
s_Eubacterium ramulus|GCF_000469345	0.86	1.00	0.93	0.0071
g_Bacteroides|13470_2_62	0.61	1.00	0.78	0.0075
s_Eubacterium biforme|GCF_000156655	0.76	1.00	0.87	0.0080
f_Porphyromonadaceae|UMGS1407	0.61	0.97	0.77	0.0094
g_Bacteroides|UMGS277	0.82	1.00	0.90	0.0101
g_Eubacterium|UMGS1529	0.62	1.00	0.79	0.0105
o_Bacteroidales|UMGS880	0.90	0.97	0.94	0.0125
o_Bacteroidales|UMGS676	0.67	1.00	0.82	0.0127
g_Bacteroides|UMGS1416	0.65	1.00	0.81	0.0137
g_Prevotella|UMGS1430	0.71	1.00	0.84	0.0161
s_Lachnospiraceae bacterium 6_1_63FAA|GCF_000209425	0.92	0.97	0.94	0.0167
s_Lascolabacillus massiliensis|GCF_001282625	0.58	1.00	0.76	0.0170
c_Alphaproteobacteria|UMGS241	0.97	1.00	0.99	0.0171
g_Ruminococcus|UMGS1882	0.62	1.00	0.79	0.0172
f_Porphyromonadaceae|UMGS211	0.59	1.00	0.77	0.0173
g_Sutterella|UMGS665	0.94	1.00	0.97	0.0180
s_Odoribacter laneus|GCF_000243215	0.68	1.00	0.82	0.0181
g_Bacteroides|UMGS901	0.62	1.00	0.78	0.0184
o_Bacteroidales|UMGS451	0.57	1.00	0.76	0.0191
f_Ruminococcaceae|UMGS1071	0.90	1.00	0.95	0.0195
g_Blautia|UMGS276	0.71	1.00	0.84	0.0209
g_Blautia|UMGS97	0.90	1.00	0.95	0.0226
g_Blautia|UMGS1359	0.67	1.00	0.82	0.0226
g_Prevotella|UMGS1007	0.60	1.00	0.78	0.0227
g_Solobacterium|UMGS1737	0.72	1.00	0.85	0.0262
g_Bacteroides|UMGS355	0.59	1.00	0.77	0.0275
g_Dysgonomonas|UMGS2	0.93	1.00	0.96	0.0276
g_Prevotella|UMGS1684	0.70	1.00	0.83	0.0276
g_Bacteroides|UMGS1534	0.59	1.00	0.77	0.0305
o_Bacteroidales|UMGS764	0.72	1.00	0.85	0.0323
f_Porphyromonadaceae|UMGS659	0.65	1.00	0.81	0.0345
g_Bacteroides|UMGS1213	0.58	1.00	0.76	0.0359
g_Bacteroides|UMGS1647	0.72	1.00	0.85	0.0370
g_Eubacterium|UMGS574	0.62	1.00	0.79	0.0381
p_Tenericutes|UMGS2065	0.72	1.00	0.85	0.0387
o_Clostridiales|UMGS977	0.61	1.00	0.78	0.0393
o_Bacteroidales|UMGS1768	0.58	1.00	0.76	0.0395
g_Bacteroides|UMGS212	0.95	1.00	0.98	0.0398
g_Eubacterium|UMGS562	0.69	1.00	0.83	0.0400
g_Succinatimonas|UMGS1092	0.65	1.00	0.81	0.0407
g_Clostridium|12718_7_39	0.92	1.00	0.96	0.0418
g_Blautia|UMGS1369	0.66	1.00	0.81	0.0423
g_Bacteroides|UMGS1265	0.63	1.00	0.79	0.0424
g_Blautia|20298_3_81	0.90	1.00	0.95	0.0446
g_Solobacterium|UMGS1017	0.62	1.00	0.79	0.0447
g_Bacteroides|UMGS1352	0.62	1.00	0.79	0.0448
g_Blautia|UMGS335	0.80	1.00	0.89	0.0454
g_Blautia|UMGS967	0.67	1.00	0.82	0.0454
g_Sutterella|UMGS767	0.81	1.00	0.90	0.0458
g_Bacteroides|UMGS1205	0.74	1.00	0.86	0.0491
s_Dysgonomonas gadei|GCF_000213555	0.67	1.00	0.82	0.0491
g_Solobacterium|UMGS695	0.59	1.00	0.77	0.0497
s_Lachnospiraceae bacterium 2_1_46FAA|GCF_000209385	0.64	1.00	0.80	0.0499

[1] Species names are formatted as beginning with their closest known taxonomy level assigned to them from s_:species, g_:genus, o_:order, f_:family, and c_:class or none when there is no assigned name for the species followed by "|" and the species identifier from the reference database.
[2] only statistically significant results (p<0.05)

Supplementary Table S7 R packages used for all the analyses described in the article. The codes were compiled based on R version 4.1.2 in RStudio.

Package	Version	Reference
abdiv	0.2.0	Kyle Bittinger (2020). abdiv: Alpha and Beta Diversity Measures. R package version 0.2.0. https://CRAN.R-project.org/package=abdiv

arsenal	3.6.3	Ethan Heinzen, Jason Sinnwell, Elizabeth Atkinson, Tina Gunderson and Gregory Dougherty (2021). arsenal: An Arsenal of 'R' Functions for Large-Scale Statistical Summaries. R package version 3.6.3. https://CRAN.R-project.org/package=arsenal
boot	1.3.28	Angelo Canty and Brian Ripley (2021). boot: Bootstrap R (S-Plus) Functions. R package version 1.3-28.
broom	1.0.0	David Robinson, Alex Hayes and Simon Couch (2022). broom: Convert Statistical Objects into Tidy Tibbles. R package version 1.0.0. https://CRAN.R-project.org/package=broom
confintr	0.1.2	Michael Mayer (2022). confintr: Confidence Intervals. R package version 0.1.2. https://CRAN.R-project.org/package=confintr
CORElearn	1.56.0	Marko Robnik-Sikonja and Petr Savicky (2021). CORElearn: Classification, Regression and Feature Evaluation. R package version 1.56.0. https://CRAN.R-project.org/package=CORElearn
cowplot	1.1.1	Claus O. Wilke (2020). cowplot: Streamlined Plot Theme and Plot Annotations for 'ggplot2'. R package version 1.1.1. https://CRAN.R-project.org/package=cowplot
cvms	1.3.4	Ludvig Renbo Olsen and Hugh Benjamin Zachariae (2022). cvms: Cross-Validation for Model Selection. R package version 1.3.4. https://CRAN.R-project.org/package=cvms
data.table	1.14.2	Matt Dowle and Arun Srinivasan (2021). data.table: Extension of `data.frame`. R package version 1.14.2. https://CRAN.R-project.org/package=data.table
DescTools	0.99.45	Andri Signorell et mult. al. (2022). DescTools: Tools for descriptive statistics. R package version 0.99.45.
dplyr	1.0.9	Hadley Wickham, Romain François, Lionel Henry and Kirill Müller (2022). dplyr: A Grammar of Data Manipulation. R package version 1.0.9. https://CRAN.R-project.org/package=dplyr
egg	0.4.5	Baptiste Auguie (2019). egg: Extensions for 'ggplot2': Custom Geom, Custom Themes, Plot Alignment, Labelled Panels, Symmetric Scales, and Fixed Panel Size. R package version 0.4.5. https://CRAN.R-project.org/package=egg
EnhancedVolcano	1.12.0	Kevin Blighe, Sharmila Rana and Myles Lewis (2021). EnhancedVolcano: Publication-ready volcano plots with enhanced colouring and labeling. R package version 1.12.0. https://github.com/kevinblighe/EnhancedVolcano
forcats	0.5.1	Hadley Wickham (2021). forcats: Tools for Working with Categorical Variables (Factors). R package version 0.5.1. https://CRAN.R-project.org/package=forcats
ggforce	0.3.3	Thomas Lin Pedersen (2021). ggforce: Accelerating 'ggplot2'. R package version 0.3.3. https://CRAN.R-project.org/package=ggforce
ggimage	0.3.1	Guangchuang Yu (2022). ggimage: Use Image in 'ggplot2'. R package version 0.3.1. https://CRAN.R-project.org/package=ggimage
ggplot2	3.3.6	H. Wickham. ggplot2: Elegant Graphics for Data Analysis. Springer-Verlag New York, 2016.
ggpubr	0.4.0	Alboukadel Kassambara (2020). ggpubr: 'ggplot2' Based Publication Ready Plots. R package version 0.4.0. https://CRAN.R-project.org/package=ggpubr
ggrepel	0.9.1	Kamil Slowikowski (2021). ggrepel: Automatically Position Non-Overlapping Text Labels with 'ggplot2'. R package version 0.9.1. https://CRAN.R-project.org/package=ggrepel
ggsignif	0.6.3	Ahlmann-Eltze, C., & Patil, I. (2021). ggsignif: R Package for Displaying Significance Brackets for 'ggplot2'. PsyArxiv. doi:10.31234/osf.io/7awm6
glmnet	4.1.4	Jerome Friedman, Trevor Hastie, Robert Tibshirani (2010). Regularization Paths for Generalized Linear Models via Coordinate Descent. Journal of Statistical Software, 33(1), 1-22. URL https://www.jstatsoft.org/v33/i01/.
gridExtra	2.3	Baptiste Auguie (2017). gridExtra: Miscellaneous Functions for "Grid" Graphics. R package version 2.3. https://CRAN.R-project.org/package=gridExtra
imbalance	1.0.2.1	Cordón I, García S, Fernández A, Herrera F (2018). "Imbalance: Oversampling algorithms for imbalanced classification in R." Knowledge-Based Systems, 161, 329-341. https://doi.org/10.1016/j.knosys.2018.07.035
indicspecies	1.7.12	De Caceres, M., Legendre, P. (2009). Associations between species and groups of sites: indices and statistical inference. Ecology, URL http://sites.google.com/site/miqueldecaceres/
infotheo	1.2.0.1	Patrick E. Meyer (2022). infotheo: Information-Theoretic Measures. R package version 1.2.0.1. https://CRAN.R-project.org/package=infotheo
jtools	2.2.0	Long JA (2022). _jtools: Analysis and Presentation of Social Scientific Data_. R package version 2.2.0, <URL:https://cran.r-project.org/package=jtools>.
kohonen	3.0.11	Wehrens R, Kruisselbrink J (2018). "Flexible Self-Organizing Maps in kohonen 3.0." _Journal of Statistical Software_, *87*(7), 1-18. doi:10.18637/jss.v087.i07 (URL: https://doi.org/10.18637/jss.v087.i07).
lares	5.1.2	Bernardo Lares (2022). lares: Analytics & Machine Learning Sidekick. R package version 5.1.2. https://CRAN.R-project.org/package=lares
lattice	0.20.45	Sarkar, Deepayan (2008) Lattice: Multivariate Data Visualization with R. Springer, New York. ISBN 978-0-387-75968-5
lavaan	0.6.12	Yves Rosseel (2012). lavaan: An R Package for Structural Equation Modeling. Journal of Statistical Software, 48(2), 1-36. https://doi.org/10.18637/jss.v048.i02
lme4	1.1.30	Douglas Bates, Martin Maechler, Ben Bolker, Steve Walker (2015). Fitting Linear Mixed-Effects Models Using lme4. Journal of Statistical Software, 67(1), 1-48. doi:10.18637/jss.v067.i01.
magrittr	2.0.3	Stefan Milton Bache and Hadley Wickham (2022). magrittr: A Forward-Pipe Operator for R. R package version 2.0.3. https://CRAN.R-project.org/package=magrittr
MASS	7.3.54	Venables, W. N. & Ripley, B. D. (2002) Modern Applied Statistics with S. Fourth Edition. Springer, New York. ISBN 0-387-95457-0
Matrix	1.3.4	Douglas Bates and Martin Maechler (2021). Matrix: Sparse and Dense Matrix Classes and Methods. R package version 1.3-4. https://CRAN.R-project.org/package=Matrix

mlr	2.19.0	Bischl B, Lang M, Kotthoff L, Schiffner J, Richter J, Studerus E, Casalicchio G, Jones Z (2016). “mlr: Machine Learning in R.” _Journal ofMachine Learning Research_, *17*(170), 1-5. <URL: https://jmlr.org/papers/v17/15-066.html>.
mltools	0.3.5	Ben Gorman (2018). mltools: Machine Learning Tools. R package version 0.3.5. https://CRAN.R-project.org/package=mltools
outliers	0.15	Lukasz Komsta (2022). outliers: Tests for Outliers. R package version 0.15. https://CRAN.R-project.org/package=outliers
parallelMap	1.5.1	Bernd Bischl, Michel Lang and Patrick Schratz (2021). parallelMap: Unified Interface to Parallelization Back-Ends. R package version 1.5.1. https://CRAN.R-project.org/package=parallelMap
ParamHelpers	1.14.1	Bernd Bischl, Michel Lang, Jakob Richter, Jakob Bossek, Daniel Horn and Pascal Kerschke (2022). ParamHelpers: Helpers for Parameters in Black-Box Optimization, Tuning and Machine Learning. R package version 1.14.1. https://CRAN.R-project.org/package=ParamHelpers
PearsonDS	1.2.2	Martin Becker and Stefan Klößner (2022). PearsonDS: Pearson Distribution System. R package version 1.2.2. https://CRAN.R-project.org/package=PearsonDS
PERFect	1.8.0	Ekaterina Smirnova and Quy Cao (2021). PERFect: Permutation filtration for microbiome data. R package version 1.8.0. https://github.com/cxquy91/PERFect
permute	0.9.7	Gavin L. Simpson (2022). permute: Functions for Generating Restricted Permutations of Data. R package version 0.9-7. https://CRAN.R-project.org/package=permute
pheatmap	1.0.12	Raivo Kolde (2019). pheatmap: Pretty Heatmaps. R package version 1.0.12. https://CRAN.R-project.org/package=pheatmap
phyloseq	1.38.0	phyloseq: An R package for reproducible interactive analysis and graphics of microbiome census data. Paul J. McMurdie and Susan Holmes (2013) PLoS ONE 8(4):e61217.
plyr	1.8.7	Hadley Wickham (2011). The Split-Apply-Combine Strategy for Data Analysis. Journal of Statistical Software, 40(1), 1-29. URL https://www.jstatsoft.org/v40/i01/.
powerMediation	0.3.4	Weiliang Qiu (2021). powerMediation: Power/Sample Size Calculation for Mediation Analysis. R package version 0.3.4. https://CRAN.R-project.org/package=powerMediation
purrr	0.3.4	Lionel Henry and Hadley Wickham (2020). purrr: Functional Programming Tools. R package version 0.3.4. https://CRAN.R-project.org/package=purrr
pwr2ppl	0.4.0	Chris Aberson (2022). pwr2ppl: Power Analyses for Common Designs (Power to the People). R package version 0.4.0. https://CRAN.R-project.org/package=pwr2ppl
R	4.1.2	R Core Team (2021). R: A language and environment for statistical computing. R Foundation for Statistical Computing, Vienna, Austria. URL https://www.R-project.org/.
rcompanion	2.4.18	Salvatore Mangiafico (2022). rcompanion: Functions to Support Extension Education Program Evaluation. R package version 2.4.18. https://CRAN.R-project.org/package=rcompanion
readr	2.1.2	Hadley Wickham, Jim Hester and Jennifer Bryan (2022). readr: Read Rectangular Text Data. R package version 2.1.2. https://CRAN.R-project.org/package=readr
readxl	1.4.0	Hadley Wickham and Jennifer Bryan (2022). readxl: Read Excel Files. R package version 1.4.0. https://CRAN.R-project.org/package=readxl
report	0.5.1	Makowski, D., Ben-Shachar, M.S., Patil, I. & Lüdecke, D. (2020). Automated Results Reporting as a Practical Tool to Improve Reproducibility and Methodological Best Practices Adoption. CRAN. Available from https://github.com/easystats/report. doi: .
repr	1.1.4	Philipp Angerer, Thomas Kluyver and Jan Schulz (2022). repr: Serializable Representations. R package version 1.1.4. https://CRAN.R-project.org/package=repr
ResourceSelection	0.3.5	Subhash R. Lele, Jonah L. Keim and Peter Solymos (2019). ResourceSelection: Resource Selection (Probability) Functions for Use-Availability Data. R package version 0.3-5. https://CRAN.R-project.org/package=ResourceSelection
rsvg	2.3.1	Jeroen Ooms (2022). rsvg: Render SVG Images into PDF, PNG, (Encapsulated) PostScript, or Bitmap Arrays. R package version 2.3.1. https://CRAN.R-project.org/package=rsvg
scales	1.2.0	Hadley Wickham and Dana Seidel (2022). scales: Scale Functions for Visualization. R package version 1.2.0. https://CRAN.R-project.org/package=scales
SIAMCAT	1.14.0	Wirbel, J., Zych, K., Essex, M. et al. Microbiome meta-analysis and cross-disease comparison enabled by the SIAMCAT machine learning toolbox. Genome Biol 22, 93 (2021). https://doi.org/10.1186/s13059-021-02306-1
sn	2.1.0	Azzalini, A. (2022). The R package 'sn': The Skew-Normal and Related Distributions such as the Skew-t and the SUN (version 2.1.0). URL http://azzalini.stat.unipd.it/SN/,https://cran.r-project.org/package=sn
stringr	1.4.0	Hadley Wickham (2019). stringr: Simple, Consistent Wrappers for Common String Operations. R package version 1.4.0. https://CRAN.R-project.org/package=stringr
tibble	3.1.8	Kirill Müller and Hadley Wickham (2022). tibble: Simple Data Frames. R package version 3.1.8. https://CRAN.R-project.org/package=tibble
tidyr	1.2.0	Hadley Wickham and Maximilian Girlich (2022). tidyr: Tidy Messy Data. R package version 1.2.0. https://CRAN.R-project.org/package=tidyr
tidyverse	1.3.2	Wickham H, Averick M, Bryan J, Chang W, McGowan LD, François R, Grolemund G, Hayes A, Henry L, Hester J, Kuhn M, Pedersen TL, Miller E, BacheSM, Müller K, Ooms J, Robinson D, Seidel DP, Spinu V, Takahashi K, Vaughan D, Wilke C, Woo K, Yutani H (2019). “Welcome to the tidyverse.”_Journal of Open Source Software_, *4*(43), 1686. doi: 10.21105/joss.01686 (URL: https://doi.org/10.21105/joss.01686).
truncnorm	1.0.8	Olaf Mersmann, Heike Trautmann, Detlef Steuer and Björn Bornkamp (2018). truncnorm: Truncated Normal Distribution. R package version 1.0-8. https://CRAN.R-project.org/package=truncnorm
tsutils	0.9.3	Nikolaos Kourentzes (2022). tsutils: Time Series Exploration, Modelling and Forecasting. R package version 0.9.3. https://CRAN.R-project.org/package=tsutils

vegan	2.6.2	Jari Oksanen, Gavin L. Simpson, F. Guillaume Blanchet, Roeland Kindt, Pierre Legendre, Peter R. Minchin, R.B. O'Hara, Peter Solymos, M. Henry H. Stevens, Eduard Szoecs, Helene Wagner, Matt Barbour, Michael Bedward, Ben Bolker, Daniel Borcard, Gustavo Carvalho, Michael Chirico, Miquel De Caceres, Sebastien Durand, Heloisa Beatriz Antoniazi Evangelista, Rich FitzJohn, Michael Friendly, Brendan Furneaux, Geoffrey Hannigan, Mark O. Hill, Leo Lahti, Dan McGlinn, Marie-Helene Ouellette, Eduardo Ribeiro Cunha, Tyler Smith, Adrian Stier, Cajo J.F. Ter Braak and James Weedon (2022). vegan: Community Ecology Package. R package version 2.6-2. https://CRAN.R-project.org/package=vegan
WebPower	0.7	Zhiyong Zhang and Yujiao Mai (2022). WebPower: Basic and Advanced Statistical Power Analysis. R package version 0.7. https://CRAN.R-project.org/package=WebPower
writexl	1.4.0	Jeroen Ooms (2021). writexl: Export Data Frames to Excel 'xlsx' Format. R package version 1.4.0. https://CRAN.R-project.org/package=writexl
